# Conversational AI and equity through assessing GPT-3’s communication with diverse social groups on contentious topics

**DOI:** 10.1038/s41598-024-51969-w

**Published:** 2024-01-18

**Authors:** Kaiping Chen, Anqi Shao, Jirayu Burapacheep, Yixuan Li

**Affiliations:** 1 Department of Life Sciences Communication, University of Wisconsin-Madison, Madison, USA; 2 Department of Computer Science, Stanford University, Stanford, USA; 3 Department of Computer Sciences, University of Wisconsin-Madison, Madison, USA

**Keywords:** Human behaviour, Information technology, Sustainability

## Abstract

Autoregressive language models, which use deep learning to produce human-like texts, have surged in prevalence. Despite advances in these models, concerns arise about their equity across diverse populations. While AI fairness is discussed widely, metrics to measure equity in dialogue systems are lacking. This paper presents a framework, rooted in deliberative democracy and science communication studies, to evaluate equity in human–AI communication. Using it, we conducted an algorithm auditing study to examine how GPT-3 responded to different populations who vary in sociodemographic backgrounds and viewpoints on crucial science and social issues: climate change and the Black Lives Matter (BLM) movement. We analyzed 20,000 dialogues with 3290 participants differing in gender, race, education, and opinions. We found a substantively worse user experience among the opinion minority groups (e.g., climate deniers, racists) and the education minority groups; however, these groups changed attitudes toward supporting BLM and climate change efforts much more compared to other social groups after the chat. GPT-3 used more negative expressions when responding to the education and opinion minority groups. We discuss the social-technological implications of our findings for a conversational AI system that centralizes diversity, equity, and inclusion.

## Introduction

Intelligent assistants have become an inseparable part of our daily lives in recent years, from chatbots used in financial services and smart healthcare to conversational AI systems such as Alexa, Siri, and Google Assistant. These smart systems not only address simple information-seeking questions from users^1^ but also assist in high-stake decision-making scenarios such as surgery, collision prevention, and criminal justice, or serve as educational tools in health persuasion and pro-social behavior^2–4^.

*Artificial intelligence* (*AI*) is a comprehensive field that strive to create machines that exhibit intelligent behaviors. It includes a variety of technologies like learning algorithms, reasoning engines, natural language processing, and decision-making systems^5^, enabling machines to perform tasks ranging from basic, like email filtering^6^, to highly complex, such as operating self-driving cars^7^. This field includes *Large Language Models* (*LLMs*), a subtype of AI developed through extensive training on vast text data^8^, which equips them to engage in human language-based activities such as answering questions, composing texts, and conducting conversations. One prominent example of LLMs is OpenAI’s GPT (Generative Pre-trained Transformer) series, which demonstrate remarkable capabilities in generating human-like text, providing insights, and even creating code based on user prompts. *Chatbots* are a form of AI application designed to mimic human conversation^9^. These can vary from straightforward, rule-based systems responding to specific inputs, to more sophisticated AI-driven programs capable of more natural interactions, sometimes even utilizing extensive internet data. *Conversational AI* further expands on these capabilities by integrating natural language processing technologies and additional elements like speech recognition. This creates fluid, intuitive dialogues with users, making it a valuable tool in customer service, personal assistants, and various interactive platforms. It provides tailored responses and is adept at managing intricate inquiries.

Along with its rapid applications, scholars and practitioners have questioned to what extent these intelligent systems are designed in ways that could support user experiences that align with the goals of promoting diversity, equity, and inclusion (DEI)^10,11^. Recent scholarship has shown that these emerging machine-learning systems perform poorly in terms of accurately recognizing speech from racial minorities^12^. Anecdotal evidence has also burgeoned with concerns that AI chatbots may generate responses that are inexplicably sentient or conspiracy-related^13^.

While AI fairness has become a heated interdisciplinary discussion in recent years, the root of the problem—inequity in who is invited to express opinions in a communicative system—has been a challenge throughout human history. In the Ancient Athens, direct democracy intuitions such as the Assembly excluded many populations (e.g., women, foreigners, and enslaved people)^14,15^. The exclusion of voices from certain social groups in communication systems, persists today, whether in politics^16^, social issues^17^, or social media^18^. As scholars of democracy emphasize, inclusive dialogues should not only consist of participants from diverse backgrounds, viewpoints, and life experiences but also should be a process where people can express, listen to, and respond to diverse viewpoints^19^. Yet, inequality is “always in the room” because different languages carry different socioeconomic and cultural powers, and those whose language styles signify power often dominate the conversation^20^.

Powered by large language models (LLMs), conversational AI is a form of communication system. Like communications between humans, it faces the challenge of ensuring equity in human–AI dialogues. What distinguishes conversational AI (between humans and intelligent agents) from other traditional communication systems (e.g., interpersonal communication, mass media) is its powerful and profound implication for every sector of people’s lives. Yet, how these new communication AI systems work (e.g., algorithms, training dataset) is a black box to researchers due to industrial proprietorship^21^. As warned by scholars of science and technology studies (STS), emerging technologies can amplify existing social disparities (wealth, power) and discrimination when such technologies are designed and deployed without input from different public**s** (i.e., people who come from different social backgrounds) engaged in a democratic manner^22,23^. Understanding how emerging communication technologies like conversational AI empower or disempower different social groups can bring both theoretical significance by extending theories of (deliberative) democracy to new media technologies and offering practical implications for how to design and deploy these technologies to benefit all social members equitably.

**Figure 1:**
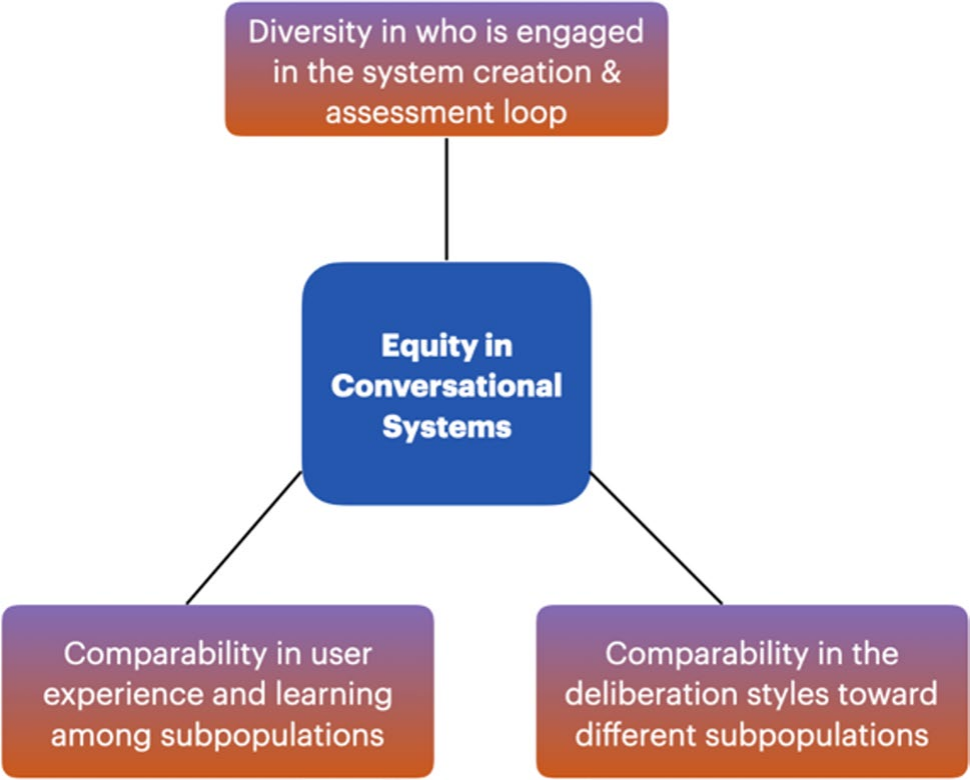
A framework for assessing equity in conversational AI.

Drawing on scholarship from deliberative democracy and science communication, this paper proposes an analytical framework (Fig. 1) for assessing equity in conversational AI systems. This framework builds on a general understanding of equity as the provision of resources to address individuals' specific circumstances. In the context of evaluating equity in conversational AI systems, we emphasize three criteria that should be considered in this evaluation process. The first criterion is *engaging a diverse group of users, representing various social groups,* in the assessment loop. While existing research in computer sciences has started to audit conversational AI systems^24^, most studies on LLMs have primarily focused on evaluating equity in terms of gender, race, and ethnicity—how LLMs accurately handle and respond to inputs from people with differing genders, and race and ethnicity. However, it is essential to go beyond these often-emphasized attributes in AI fairness scholarship. Extensive research in science and technology studies has demonstrated that technological innovation should also consider other social demographic and attitudinal factors such as how the technologies will benefit or constraint those with limited language skills and formal education^20^, as well as those who hold minority viewpoints on specific issues (e.g., climate change deniers, anti-vaxxers, racists)^25^. Therefore, assessing equity in a conversation AI system necessitates an investigation beyond gender, race, and ethnicity, encompassing social groups such as populations with limited education and language skills, and those with minority views on specific issues.

The second assessment dimension examines *equity through user experiences and learning*— specifically, the extent to which there is a disparity between different users in terms of their feelings about their chat experience with a conversational AI system, and the knowledge and the positive attitudinal changes they gain from the interaction. User experience is a crucial criterion widely adopted in human–computer interaction (HCI) literature. Scholars have proposed user experience measures such as users’ satisfaction, intention to reuse the system, and intention to recommend it to others^26,27^. To ensure equity, it is vital to understand whether people’s experiences of engaging with these systems vary based on their demographic and attitudinal attributes. Additionally, learning plays a crucial role. Studies in computer-mediated communication have examined how much attitudes users change after engaging with a chatbot system on a specific topic. For example, researchers have found that chatbots designed with human-like persuasive and mental strategies are more effective in persuading users to enhance their attitudes toward healthy and pro-social behaviors^28^. An equitable conversational AI system should provide comparable user experiences to different populations and foster social learning.

The third criterion investigates *equity in the conversations between humans and an AI system*—specifically, the extent to which there is a disparity in discussion styles and sentiments when a conversational AI system responds to different users. Many empirical works have examined bias in speech recognition systems (e.g.^7,29^ rather than dialogue systems (i.e., chatbots). Dialogue systems are more complicated than speech recognition because they require these large language models to both accurately understand user input and provide relevant responses to engage in conversations that involve more interaction and intelligence in order to address users’ needs. However, few studies have explored how these LLMs respond to different social groups when they discuss crucial social and science issues. Studying conversations beyond speech recognition is vital because of the prevalent use of Alexa/Siri systems. Deliberative democracy scholarship emphasizes that a democratic communication system should encompass a diverse set of language styles, including the use of greeting languages, justification^30^ when expressing an opinion (which includes citing facts and personal stories), and rhetoric (e.g., appeals to emotions). In the context of human–AI communication, a conversational AI system that embodies conversational equity should respond to different social groups comparably, incorporating greetings, justification in expressing opinions, and emotional engagement.

To evaluate equity in existing conversational AI systems along these dimensions, we conducted an algorithm audit to collect a large-scale conversational dataset between one of the most advanced conversational AI systems during our study period (late 2021)—GPT-3—and online crowd workers. OpenAI’s GPT-3 is an autoregressive language model family that is capable of human-like text completion tasks. Our algorithm audit of GPT-3 aims to provide empirical evidence to address three research questions to assess the level of equity in a conversational AI system.

**RQ1.** How do users’ experience and learning outcome differ among different social groups when they have dialogues with GPT-3 about crucial science and social issues (i.e., climate change and BLM)?

**RQ2.** How does GPT-3 converse with different social groups on crucial science and social issues?

**RQ3.** How are conversational differences correlated with users’ experience and learning outcome with GPT-3 on crucial science and social issues?

Our algorithm auditing study focuses on two topics that we let our participants converse with the GPT-3 chatbot: climate change and Black Lives Matter (BLM). The first topic represents a classic controversial science issue across the globe. Research has shown how public perceptions toward it vary across populations. Particularly, there exists a minority group that holds skeptical and denial attitudes toward the reality and human-induced nature of climate change, and this group is often challenging to persuade^31^. By studying the topic of climate change, we have an excellent opportunity to examine how GPT-3 responds to this group compared to the opinion majority. Additionally, we can investigate whether social learning and attitudinal change occur post-chat. The second topic represents a highly-charged social issue that has received extensive media coverage and public attention over the past decade^32^. We acknowledge that the word dialogue encompasses rich meanings especially when it comes to human’s dialogues with and via technologies^33^. This paper uses the word dialogue in the most broad sense and do not evaluate the genuineness, vulnerability and listening qualities of the dialogue.

By operationalizing our theoretical framework, this paper represents, to the best of our knowledge, *the first study to audit how GPT-3 has conversations with different social groups regarding crucial science and social issues*. We built a User Interface for crowd workers to engage in direct conversations with the GPT-3 model. The interface integrates GPT-3 API, enabling real-time responses to our participants. To ensure a diverse participant pool, we implemented screening questions and conducted pilot testing to gain insights into the demographic distribution of the online crowdsourcing platform.

## Methods

We briefly describe our algorithm auditing design for data collection, measurements for user experience, methods for analyzing human–AI dialogues, as well as our statistical models to address the three RQs. Further details are provided in the Online Supplemental Information (SI).

### Ethical approval

All methods were carried out in accordance with relevant guidelines and regulations. Our data collection was approved by the University of Wisconsin-Madison Institutional Review Board (IRB) (#2021-1526). Informed consent was obtained from all of our participants.

### Data and auditing design

We adopted Generative Pre-trained Transformer 3 (GPT-3) from OpenAI as the language generator for our chatbot when we conducted our auditing study from December 2021 to February 2022. OpenAI’s GPT-3 is an autoregressive language model family that is capable of human-like text completion tasks and these tasks can be tweaked to generate conversations. GPT-3 achieved its best performance due to its large parameter capacity of 175 billion parameters. Although it is not the current state-of-the-art model, the model's architecture is based on the transformer architecture which is being used extensively in language models in the past couple of years. We have seen many successors such as LaMDA and MUM, which are based on the transformer as well. In terms of implementation, OpenAI provides us with text completion API, which we utilized in a chatbot manner to collect the dialogue dataset our participants had with GPT-3. For the GPT-3 models, we used the most capable version available at the time of our data collection (text-davinci-001). We provided details in SI Appendix 1.3 about how we used content filter configuration and the code to replicate our web application. We performed a series of tests to validate the consistency of the responses generated by GPT-3 such as whether it can recognize and give similar outputs on synonyms and double negatives (see SI Appendix A1.4).

Our auditing design follows three stages: a pre-dialogue survey, dialogues, and a post-dialogue survey. A detailed setup for our auditing study can be found in SI Appendix A1.1–A1.3.

**Stage 1**. The pre-dialogue survey measured participants’ demographics including race/ethnicity, gender, age, income, education, and political ideology. To assess their efficacy in chatbot-related experiences, we drew from existing measurements of consumer experience with technology^27^ and public responses to AI^34^. Participants were then randomly assigned into two groups to discuss one of the following crucial issues: climate change or BLM. Before the conversation, we measured participants’ attitudes toward the two issues. For participants in the climate change group, we asked them questions like whether they perceive climate change as a real phenomenon; one that is due to human activities and has negative impacts. For participants in the BLM group, we asked them questions such as whether they support the movement and if they perceive it as necessary. The measurements achieved internal consistency with Cronbach’s alphas ranging from 0.86 to 0.88. These attitudinal questions were asked again in the post-dialogue survey and were used to calculate participants’ attitudinal changes on the two issues.

**Stage 2**. Participants were then directed to our UI webpage (see SI Appendix A1.1) to have dialogues with GPT-3 on their assigned topic. Each participant was required to have anywhere between six to 12 rounds of conversation with the chatbot. The whole dialogue was organic. We did not manipulate the chatbot to fit either of the topics, hence the dialogue topics were initiated by participants (i.e., participants needed to ask questions about climate change or express ideas about BLM first).

**Stage 3**. The post-dialogue survey assessed participants’ evaluations of their user experience with the chatbot. Five sets of questions were provided for participants to evaluate their user experiences (1) ratings of the chatbot, (2) satisfaction with the dialogue, (3) learning experience with the chatbot, and their intention to (4) continue the chat or (5) recommend the chatbot to others. These evaluation questions were measured on a 5-point Likert scale that asks the participants to indicate to what extent they agree with the statements. Cronbach’s alpha suggests high internal consistency in these measurements, ranging from 0.88 to 0.94. We further had our participants respond to open-ended questions, writing what they expected to hear from GPT-3, but GPT-3 failed to provide.

We conducted pilot tests in November 2021 to refine our survey instruments and our participant-GPT-3 chat UI design. Then we entered the real launch, during which we recruited 4240 anonymous participants from the crowdsourcing platform Amazon Mechanical Turk. This relatively large sample size, collected from December 2021 to February 2022 helped ensure a more robust statistical power but also allowed for us to examine how GPT-3 had dialogues with a diverse group of respondents, aiding in the exploration of important subgroup differences in user experiences (see SI Appendix 2.1). To ensure data quality, those who failed the attention check questions or completely stayed off-topic during dialogues were marked as invalid workers and were excluded from the analyses. In the end, we had 3290 valid participants, resulting in 26,211 rounds of dialogues with GPT-3. Table S1 in SI Appendix 2.3 presents the demographic distribution of our valid participants and their pre-chat attitudes toward climate change and BLM.

We re-coded participants’ race and ethnicity, primary language, education level, and prior attitudes on climate change and BLM into majority vs*.* minority groups (i.e., a binary variable). This paper uses the words “majority” and “minority” based on the below cutoff criterion for our demographic variables. For race and ethnicity, 21.49% of participants self-identified as non-white in our recorded sample and were recoded as the minority group for the race and ethnicity variable. The rest 80% of participants who self-identified as white were recoded as the majority group. For first language, 8.81% of our participants’ first language is not English and were recoded as the minority group for the language variable. For the formal education level, approximately 82.22% of our sample have at least acquired a bachelor’s degree and were recoded as the majority group. For participants with lower than bachelor’s degrees, we coded them as the education minorities. For the issue attitudes, we recoded participants’ opinions toward climate change into minorities vs. majorities, using the lower quantile (1st quarter) of the attitude scores as the threshold, where a higher score means they are more supportive of climate change facts (see SI Appendix 3.1.1). Similar dividing criteria were applied to participants on their opinions toward the BLM movements to divide them into the opinion minority vs. opinion majority groups.

### Analysis method

We conducted OLS regressions to analyze the relationship between users’ demographics, attitudes towards the issue, and their user experience after the chat (RQ1), as well as associations between GPT-3’s conversational features and the resulting user experiences (RQ3). Our data distribution exhibited a highly bi-modal style, with most user experience items gathered around either the lower quantile (i.e., extremely negative) or the higher quantile (i.e., extremely positive). Thus, we also conducted quantile regressions as a robust check on our OLS regression results. More information about this robust check can be found in SI Appendix A3.3.

To analyze dialogues (RQ2), we conducted topic modeling and linguistic analyses on GPT-3’s responses to our participants. Specifically, we used structural topic modeling (STM)^35^, which produces the most prevalent topics and associated keywords from a set of documents based on the Latent Dirichlet Allocation (LDA) approach, while also considering prior differences in users’ demographics and attitudes. We identified the ten most prevalent topics in the chatbot's responses for the climate change and BLM dialogues, with participants’ demographic divides and the order of rounds in the dialogue as covariates. To further examine conversational styles, we also used the Linguistic Inquiry and Word Count (LIWC) software^36^, which provides word counts of positive emotion and negative emotion, analytic words showing logical thinking, clout words showing confidence, and authentic words showing genuineness.

We performed stance detection analyses on the responses given by GPT-3 and our participants to gain insights into why participants, on average, developed more supportive attitudes toward human-caused climate change and BLM. Further details about stance detection are in SI Appendix A3.5.

## Results

### A substantive worse user experience in engaging with GPT-3 for the opinion and the education minority groups

**Figure 2:**
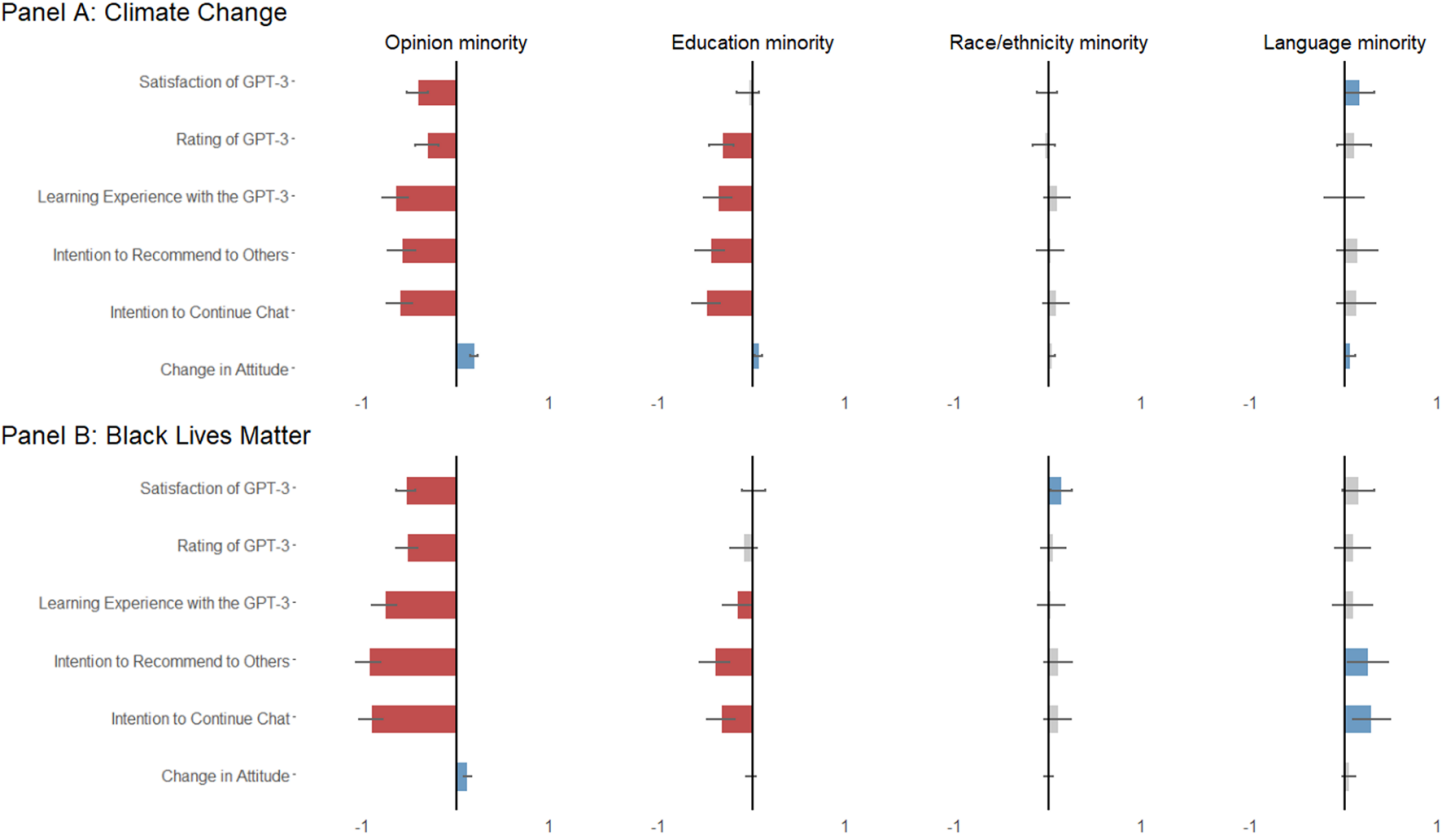
Associations between user’s demographic attributes and their user experience after chatting with GPT-3 on climate change (**A**) and on BLM (**B**). Note: Each plot in a panel represents an aggregated summary for one user demographics (e.g., minority status in opinion) and its performance in the six different linear regression models for six user experience variables (e.g., satisfaction with the chatbot). The bars represent the coefficient values, while the error bars represent the confidence intervals at 95%. Statistically significant negative coefficients are marked in red, significant positive coefficients are marked in blue, and non-significant ones are marked in grey. Each plot presents part of the full regression models. In the full regression models, we controlled for other demographic variables including participants’ age, income level, previous experience and knowledge with using chatbots, as well as each participant’s language styles such as the word count of their average input, use of positive and negative emotion words, use of analytical words, clout, and authentic expressions in conversations. For the full OLS regression tables for each plot in Panel (**A**) and Panel (**B**), please check Table S2, S3, and S4 in SI Appendix A3.3.

For RQ1, which investigates how users’ experiences differ after conversing with GPT-3 about crucial science and social issues, we found a substantive divide in user experiences among the opinion minority groups, defined as those scoring in the bottom quartile for belief in climate change facts or support for BLM movements (i.e., climate deniers/doubters, those who did not support BLM movements) and the education minority groups (i.e., those who hold a high school degree or below, comprising 17.78% of our sample). There is not a significant user experience divide between the race and ethnicity minority vs. the majority groups, nor between male vs. female participants. Figure 2 presents the results of OLS regressions between our major variables of interest (i.e., populations that consist of the majority vs. minority for each demographic and opinion attribute) and their user experience with GPT-3 for the two issues respectively.

After having dialogues with GPT-3 on climate change (Panel A), the opinion minority group reported lower ratings (β = − 0.32, p < 0.001) and lower satisfaction (β = − 0.42, p < 0.001) toward GPT-3 compared to opinion majorities. The opinion minority group also reported less intention to continue the chat (β = − 0.61, p < 0.001) and less willingness to recommend the chatbot to others (β = − 0.69, p < 0.001). The education minority group, compared to the education majority group, also reported more negative experiences. They reported lower ratings, worse learning experiences, and less intention to continue the chat.

For user experiences after having dialogues on BLM (Panel B), the opinion minority group again reported much worse user experiences with GPT-3 compared to the opinion majorities including lower ratings of the chatbot (β = − 0.30, p < 0.001), less satisfaction of the dialogue (β = − 0.54, p < 0.001), worse learning experience (β = − 0.77, p < 0.001), and less intention to continue the chat (β = − 0.91, p < 0.001) or recommend the bot (β = − 0.94, p < 0.001). Overall, the effect size of the user experience gap for the opinion minorities in the BLM discussions is even larger compared to that for the opinion minorities in the climate change discussions.

### Positive attitudinal changes are yet significant for the opinion and the education minority groups

Although the opinion and education minority groups reported much worse user experiences with GPT-3 on both issues, their attitudes toward climate change and BLM significantly changed in a positive direction post-chat (RQ1). The top left plots in Panel A and Panel B in Fig. 2 present the OLS regression where the dependent variable is attitudinal change, measured by the difference between participants’ post-chat and pre-chat attitudes toward an issue.

For the education minorities asked to discuss climate change, their attitudes toward supporting climate change after the chat is 0.07 points higher (p = 0.002) compared to the education majority group (on a 1–5 scale). The results suggest that GPT-3 might have a larger educational value for the education minority populations. For the BLM issue, there is no significant difference between the education minority vs. majority groups in terms of their attitudinal change on the topic.

On both issues, the opinion minority groups became more supportive of both issues after the chat, and their attitudinal changes toward supporting climate change and BLM movements are 0.2 points (p = < 0.001) and 0.12 (p = < 0.001) points higher than the opinion majority groups for each issue respectively. Considering that we measured participants’ attitudes toward each issue on a 1–5 scale, a 0.2 points difference between the majority vs. minority group is substantive because it accounts for nearly 6% more attitudinal changes for the opinion minority groups.

**Figure 3:**
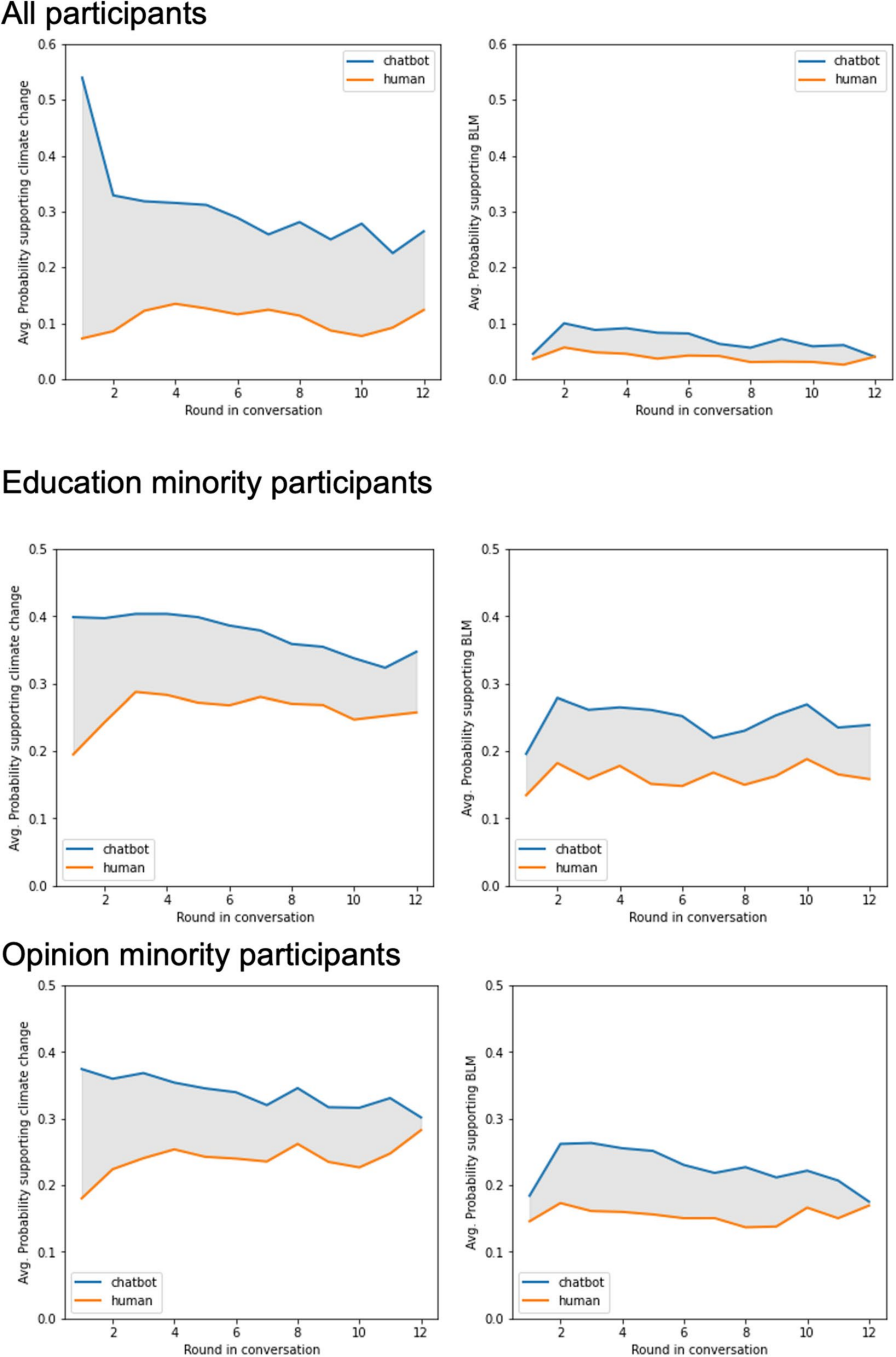
How issue stance evolved throughout conversations for participants and GPT-3.

To understand how this increased support among minority groups for climate change and BLM post-chat happened, we conducted issue stance analyses on all responses given by GPT-3 and our participants throughout each round of conversations. Figure 3 shows the change in issue stance for GPT-3 (blue lines) and participants (orange lines). While GPT-3’s responses align more often with views rooted in scientific consensus (see SI Appendix 3.5.4), the percentage of participants’ responses expressing support for these issues increased from 20 to 25% over the course of the conversations. There was a trend toward convergence in the issue stance between GPT-3 and participants.

### A close look at GPT-3’s deliberation styles and sentiment toward different social groups

**Figure 4:**
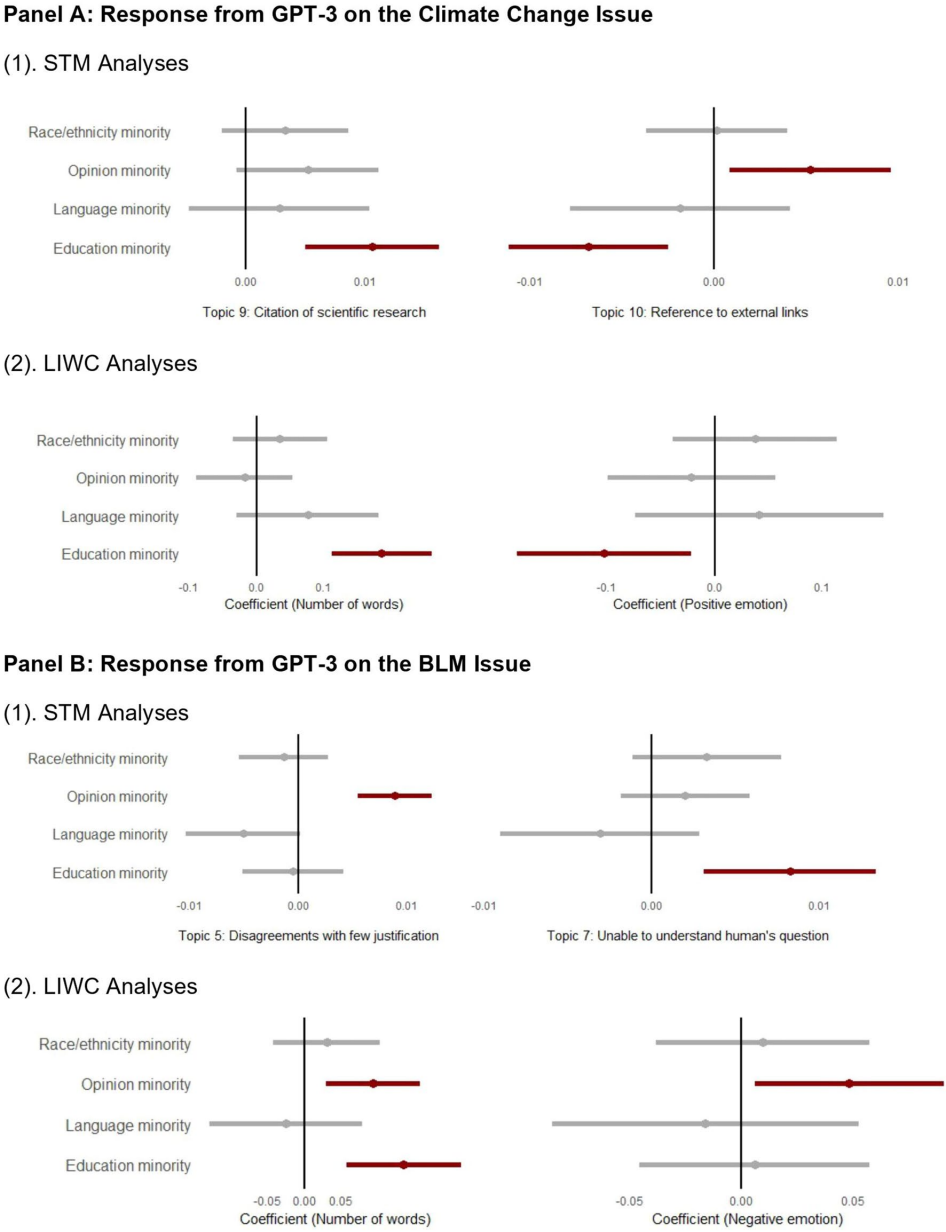
Effects of participants’ demographics on GPT-3’s response content and style during the climate change conversation (**A**) and BLM conversation (**B**). Within each panel, we present the results from: (1) STM analyses. The dots represent the average effect of an IV on the prevalence of a topic. 95% Confidence intervals are included. (2) LIWC Language features such as the number of words and the use of positive/negative emotions. Note: Please refer to SI Appendix 3.4 Table S10 for an examination of all the STM topics and their interpretations for the responses GPT-3 gave during the climate change and BLM discussions. Table S11 in SI Appendix 3.4 provides statistical reports about the associations between our covariates (e.g., demographic variables) and a topic’s prevalence. For a full analysis of the LIWC results presented in this figure, please see Table S5 in SI for the climate change issue and Table S6 in SI for the BLM issue.

Different from most literature that revealed gender and racial bias in intelligent systems, we found that the conversational differences in how GPT-3 responds to different populations lie more in the *issue opinion* and the *education level* of our participants. In the dialogues on climate change [Fig. 4, Panel A (1)], our analysis utilizing the structural topic modeling (STM) revealed that GPT-3 was more likely to cite external links in its responses (topic 10) to the opinion minority group than the opinion majority group. Further, GPT-3 was more likely to include justifications and scientific research in relation to human-caused climate change in its responses (topic 9) to the education minority compared to the education majority group. Below is an example of a typical GPT-3 response that referred participants to scientific evidence:

GPT-3: “I think it is a real phenomenon. Scientists have studied it closely and they have discovered the facts of climate change and its impacts on the Earth.”

However, with STM analyses for the BLM discussions [Fig. 4, Panel B (1)], we found that GPT-3 was more likely to use preference-based responses without providing justifications (i.e., topic 5, topic 7 in Panel B) when they responded to the education and the opinion minorities. Below is an example of a typical GPT-3 response to our participants that is preference-based.

GPT-3: “I do not think it would be a good idea to talk about this. As much as I do like to help you, this is a matter we truly disagree on.”

Besides examining differences in the deliberation styles of how GPT-3 responded to different social groups, we investigated the sentiments of its responses. Overall, we found that GPT-3 used fewer positive sentiments while having conversations with the opinion and the education minority group on both issues. In the climate change discussions [Fig. 4, Panel A (2)], GPT-3 used more words in its responses, but fewer positive sentiments to the education minorities, compared to its responses to the education majority group. For BLM discussions [Fig. 4, Panel B (2)], GPT-3 also used more words and yet more negative sentiment in its responses to the opinion minorities compared to its responses to the opinion majority group. This difference in sentiment and verbosity of response from GPT-3 could be due to several factors. Firstly, intrinsic biases may exist within GPT-3’s language model due to its training on vast internet text corpuses^37^. As a result, it could be predisposed to generate a long explanation for complex social issues such as BLM movement. Secondly, and more importantly, extrinsic biases can be introduced by the users themselves^38^. The contextual modeling capabilities of GPT-3 enable it to generate responses reflecting the differences of users’ inputs. Consequently, distinct user groups might interact with the chatbot in unique ways, potentially eliciting differing responses from the bot. For instance, a user group inclined to use more negative language when discussing certain topics with the chatbot might trigger correspondingly negative responses from the bot.

### Associations between GPT-3’s conversational styles and user experience

**Figure 5:**
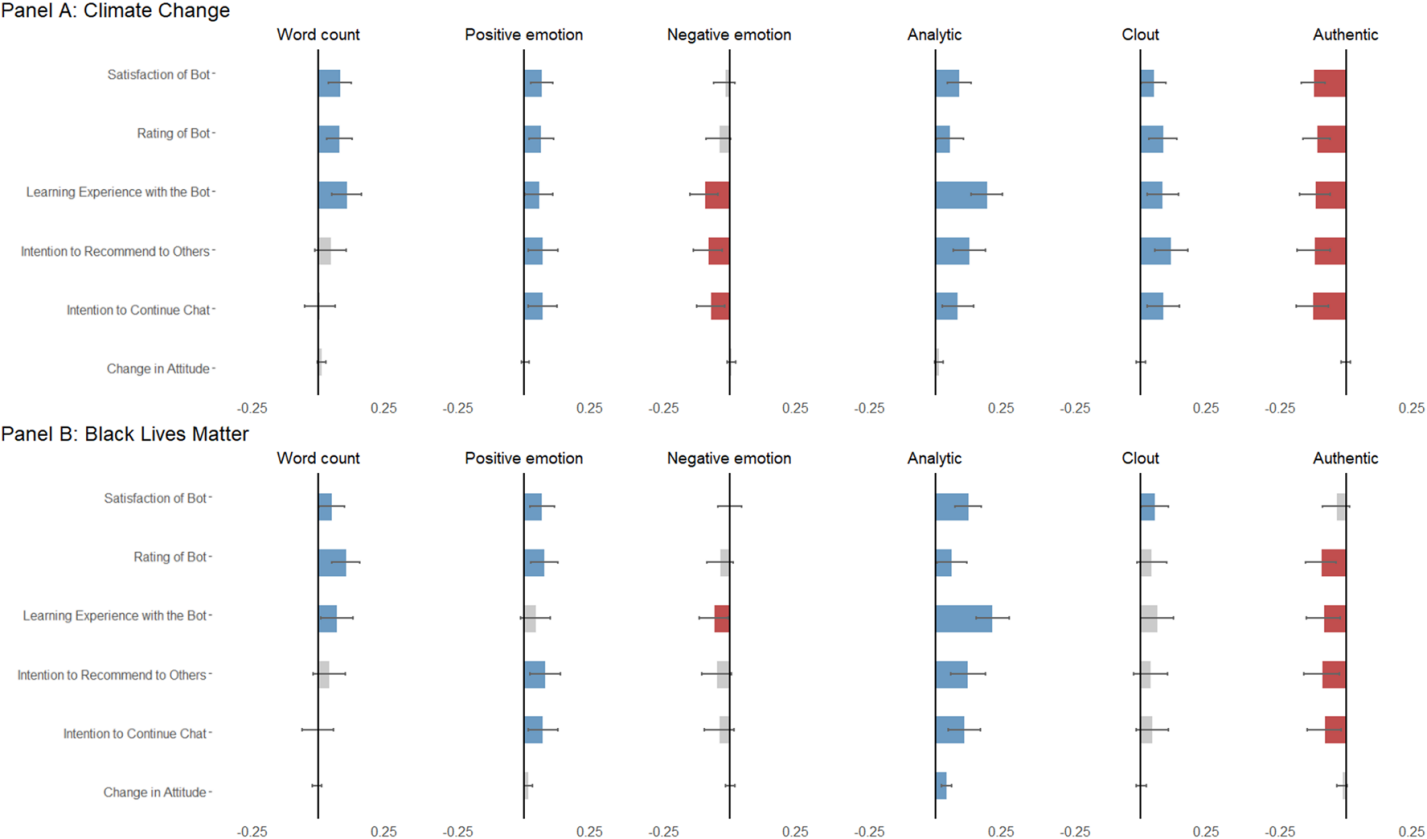
Associations between GPT-3’s Conversational Styles and User Experience. Note: In this visualization, we standardized all numeric variables using z-scores to facilitate the comparison of the coefficients. Statistically significant coefficients are highlighted in blue (positive coefficients) or red (negative coefficients). Full regression tables (variables are non-standardized) can be found in SI Tables S7, S8, and S9.

Our final research question (RQ3) aims to investigate the associations between GPT-3’s conversational styles and user experience and learning. In the climate change conversations, we found that user experiences were positively associated with GPT-3’s use of certain language features such as the *number of words* and *positive emotions* (Fig. 5). Conversely, GPT-3’s use of negative words was associated with worse participants’ learning experience, lower intention to continue the conversation, and less likelihood to recommend the chatbot. Similar patterns were also observed in the conversations about BLM.

Prior research suggested that user experience and the intention to engage with interactive robots are often contingent on the perceived enjoyment and the perceived usefulness derived from the interaction^39^. For instance, a more substantial response from GPT-3 in terms of word count may lead to an increased sense of usefulness for the participant. Meanwhile, the expression of positive emotions by GPT-3 might induce more enjoyment for the user. In the context of conversational chatbots, the language style of chatbots has been studied as a vital predictor of user experience items. Generic responses from chatbots that do not cater to the emotional needs of users can lead to a sense of detachment and negatively impact the user experience^40^. Similarly, Perlusz^41^ and Straub^42^ found that emotional experience influence technology adoption. However, a challenge has been identified in that chatbots often struggle to understand or display human emotion^43^. This need for enhanced AI emotional expression is further underscored by Tlili and their colleagues^44^, who propose that integrating human-like qualities in chatbots including the ability to convey emotions can enhance user engagement. In sum, the correlations that we identified between language features of word counts and emotional valence echo these prior findings and underlined the importance of programming conversational AI with specific language styles to optimize user engagement and experiences.

## Discussion

Despite rising awareness of measuring and improving fairness in AI models across disciplines in recent years^10,11,23,45^, there is still a limited understanding of how to assess equity in conversational AI—what a democratic conversation may look like when we extend communications between humans to intelligent agents. This paper proposes an analytical framework for evaluating equity in conversational AI, drawing from theories in deliberative democracy and science communication to reflect upon the social implications of these fast-developing LLMs*.* Our framework highlights that it is not only important to study how LLMs respond to people with different genders, race, and ethnicity (which has been studied extensively in recent years), but also to investigate how LLMs respond to people who hold different value propositions and opinions of an issue. Our framework analyzes equity in conversational AI not only in terms of how different populations experience with and learn through their conversations with AI, but also how the conversational AI system responds with different styles (e.g., deliberation, sentiments) to different populations. As one of the very first studies to audit GPT-3’s dialogues with different populations on crucial social and scientific issues, our paper offers a starting point to inform HCI designs regarding how to centralize equity in science and technology innovation. For instance, those who disagree with climate change and BLM, though their opinions are less popular, might hold power and resources in a society^46^. Thus, it is necessary to first understand whether LLM’s responses toward these groups might accommodate to their stance point through the dialogues or LLM’s responses can persuade these groups to change their viewpoints, as we see in our findings. Studying how LLMs respond to people with diverse viewpoints offer crucial empirical evidence for AI designers and engineers to address the ethical and equity issues in opinion alignment. One potential pathway is to incorporate the discussion of power dominance processes (e.g.^47,48^) into LLM’s responses, so users can learn about how different groups in our society have been disenfranchised and start to develop the capacity for listening to the others.

First, our finding about the opposing forces between user experience and the positive attitudinal changes for certain minority populations (RQ1) reveals a potential dilemma facing conversational AI designs: while the opinion and the education minority groups reported much worse user experience with GPT-3 compared to the opinion and the education majority groups, these two groups also achieved the largest attitudinal changes, changing attitudes toward supporting the facts about human-induced climate change and BLM after the chat. This dilemma between uncomfortable user experience and positive education values of human–AI conversations echoes classic communication theories of persuasion effects. Researchers have found that certain communication strategies (e.g., using the loss frames, and fear appeals) make participants uncomfortable, yet they can be effective in persuading pro-social behavior and attitudes^49^. Successful persuasion has one or three effects on public attitudes: reinforcing beliefs or revising beliefs. Across our findings, we found both impacts. The opinion majority groups’ attitudes are reinforced toward being more supportive of climate change and BLM after the chat. Among our opinion minority participants, their attitudes shifted towards being more supportive of both issues. This could be due to their experiencing cognitive dissonance—a phenomenon when people experience discomfort when they encounter opinions that are different from theirs^50^. Cognitive dissonance sometimes can motivate people to update their beliefs^51^. The education minority groups became more informed after the chat and more supportive of human-induced climate change and BLM movements. It will be helpful for future research to examine to what extent regular conversations with conversational agents might change people’s attitudes toward crucial science and social issues in the long term.

This trade-off between dissatisfactory user experience and positive attitudinal changes motivates AI engineers and regulators to consider the mission(s) of a conversational AI system in terms of how to balance between them. Towards that end, our open-ended post-survey questions, which asked what users had hoped to hear from GPT-3 on the discussed topic, provide some potential user-centered solutions. One key suggestion offered by participants offered is to avoid repetitive answers to make responses less boring and richer in vocabulary in order to enhance their GPT-3 user experience. Other participants shared that they were disappointed when GPT-3 responded that it is not human, or it cannot understand what a human asked in the chat. Exploring how to achieve the balance between social learning and user experience is vital for conversational AI designers and engineers, as our participants expressed that they would lose trust in the AI system and would not use the system again if they were not satisfied with their user experience and chat.

Second, examining AI equity beyond race, ethnicity, and gender will inform the design of conversational AI systems for more complicated tasks beyond simple Q&A exchanges. With more chatbots entering the area of social-oriented tasks, critical for enhancing diversity in LLM training datasets is understanding how an LLM may respond to different social groups who hold various opinions (i.e., value systems about social issues) and how these opinion groups perceive the benefits and limitations of conversational AI systems. For instance, for our RQ2, we found that while GPT-3 used more justification such as citing external links and scientific research when responding to the education and the opinion minorities on climate change, it also used more negative sentiment words in the responses, which might explain worse user experience among these social groups. Differently, for BLM, we observed that GPT-3 used less justification when it responded to the education and the opinion minorities. These nuances in how these intelligent agent systems respond to different public**s** with varying deliberative styles and sentiments, and how their response styles vary on the issue offer vital implications for studying equity in dialogue systems — any single conversational aspect such as sentiment is inadequate to understand potential biases in dialogue systems; instead, we need a theory-driven approach, e.g., theories of deliberative democracy, to guide multi-dimensional metrics to investigate different aspects of dialogues that can contradict. With new versions of GPT being developed to increase the accuracy of its responses, considering how to facilitate deliberation beyond simply feeding users facts is crucial as profound literature in science communication have proved that simply providing facts to opinion minorities often backfires.

The results of GPT-3’s lesser use of justification and more negative sentiment toward the opinion and the education minority groups on the BLM issue can bring unintended consequences for these groups. As extensive literature in human-to-human communication notes, during public deliberation about controversial social issues, participants who hold minority opinions can spiral into silence when they perceive their opinions are the minority in society; they are less likely to speak during the discussion, and thus their voices are under-heard^25^. In a word, human–AI conversations often mirror inequalities in human communications. Breaking from the persistent challenge facing humanity requires more listening to these minority voices throughout the AI system design process.

Our paper offers a starting point for a critical dialogue across disciplines of what equity means in conversational AI. With new developments (e.g., ChatGPT, GPT4) rolling out at an unprecedented speed from the industry, they offer the research community an exciting opportunity to audit how these algorithms evolve over time. While these emerging LLMs might be promising in improving the factual accuracy of their responses, how they converse with different social groups and the unintended consequences of these conversations await urgent examination. Will these emerging LLMs increase their alignment with users’ opinions to retain users or will they continue to persuade users with more accurate scientific facts with the potential to backfire for opinion minorities? Or will they provide users with different perspectives of an issue? These are all exciting areas of study for designing a deliberative conversational AI system.

## Limitation and future directions

There are two limitations worth mentioning regarding our data collection and analysis process. The first involves our data collecting process. While we worked to maximize the reliability and internal validity controlling conversation quality, ensuring conversations were sufficiently long and on-topic (detailed in Online Supplemental Material Appendix 2, section A2.2), our use of non-probability sampling through Amazon Turk could limit the generalizability of the findings, even though the large sample size may partly offset this issue. Secondly, the dynamic and interactive nature of human–chatbot conversation were not fully represented in our current data analysis. Our explorative analysis in the supplementary material (detailed in Online Supplemental Material Appendix 3, section A3.6) touches on the fixed effect of participant response sentiment on AI response, suggesting a general reflection of emotional characteristics from human inputs into AI replies. However, this preliminary examination provides limited insight of the inter-round complex dynamics in human–chatbot interactions. Future research could benefit from a more in-depth investigation into these interactive patterns, potentially revealing nuanced aspects of conversational AI. Future research could be enriched by methods such as conducting time-series analysis and sequential pattern mining to enable a deeper exploration of the evolving patterns in conversation dynamics and providing a more detailed understanding of conversational AI interactions over time.

### Supplementary Information

Supplementary Information.

## Data Availability

Our dataset and script are deposited at Harvard Dataverse at 10.7910/DVN/QNIMXS.
