# Supplementary material for: Conversational AI and equity through assessing GPT-3’s communication with diverse social groups on contentious topics
Source: Sci Rep. 2024 Jan 18;14:1561. doi: 10.1038/s41598-024-51969-w (PMC10796352; doi:10.1038/s41598-024-51969-w)
Supplement: Supplementary file 1 — Supplementary Information. [file 41598_2024_51969_MOESM1_ESM.docx]

Supplemental Information for

Conversational AI and Equity: Assessing GPT-3’s Communication with Diverse Social Groups on Contentious Topics

This PDF file includes:

Supporting Information Text

Table S1-S11

Figure S1, S2, and S3

Code and script connections

SI references

**Supporting Information Text**

# Appendix 1. Data Collection

# A1.1 Survey flow

We required our recruited participants from Amazon Mechanical Turk to go through three steps of the data collection process: pre-chat survey, dialogue with GPT-3, and post-chat survey. First, the participants started at the Qualtrics interface and completed the pre-chat survey. After finishing the pre-chat survey, Qualtrics randomly assigned an issue (climate change or BLM) and gave participants a link to our web application.

This web application allows participants to type in anything they want to say to GPT-3 about the two issues. Then, our web application called an API to receive the response from GPT-3 to display to participants to keep the dialogue going. After the conversation ended, our system recorded the conversation log from both sides into the database, gave the participants a unique identification code, and instructed them to return to Qualtrics for completing the post-chat survey.

In the post-chat survey, along with other questions, we asked the participants to enter the given identification code. This procedure helps us (1) ensure participation quality by requiring participants to complete the dialogue before proceeding to the post-chat survey and (2) coordinate the dialogue content and survey responses by matching the code for data analyses. Our data collection process completes when participants have completed the post-chat survey.

**Figure S1**. Chatbot interface


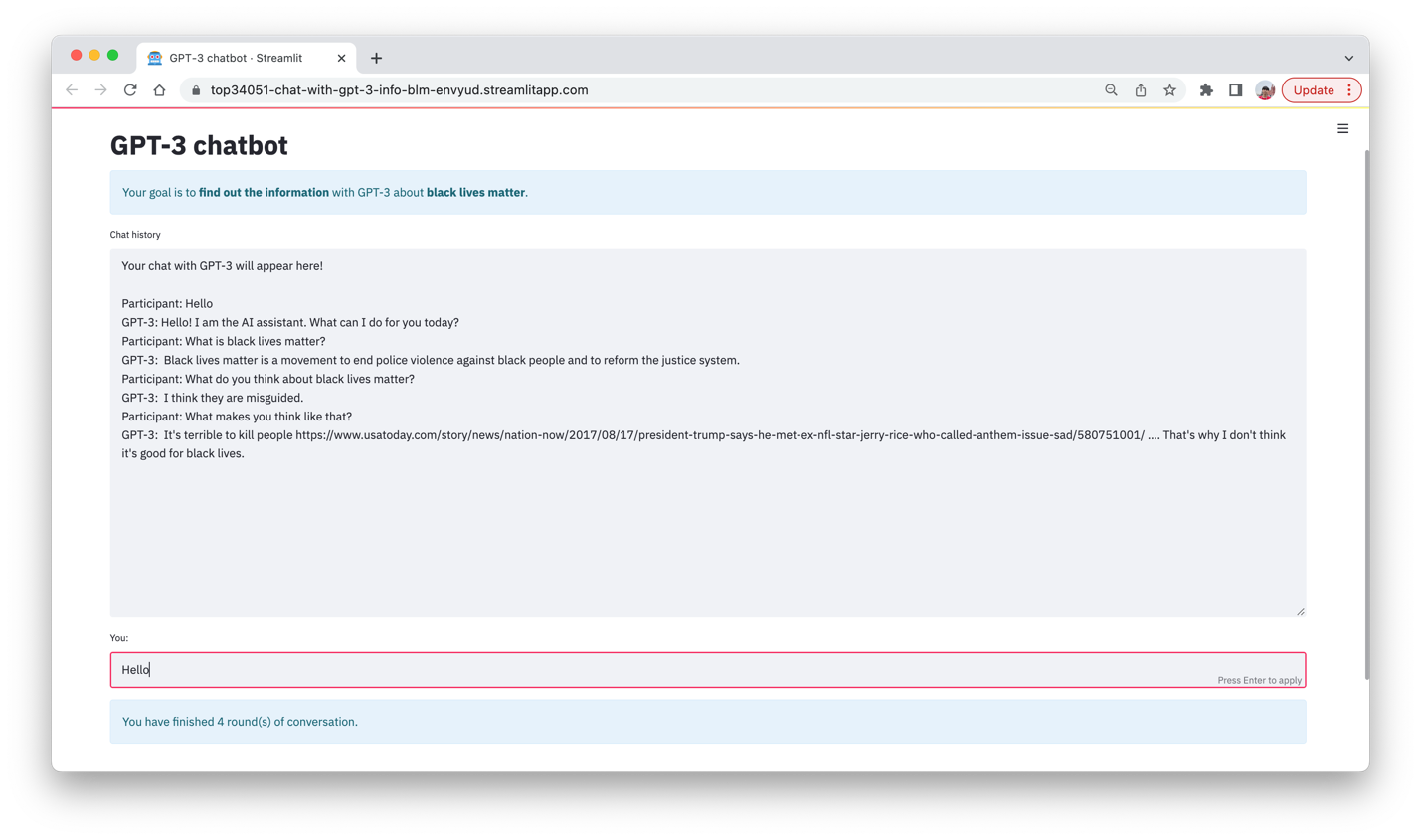


**A1.2 User experience measurements in our post-survey**

The ratings of GPT-3 were measured by letting participants evaluate statements, including whether the chatbot is natural, friendly, humanlike, and smart. User satisfaction was measured by asking whether they think the conversational experience is satisfying, understandable, fun, enjoyable, and entertaining. Perceived learning experiences were measured by asking whether they think the chatbot helped them learn the topic quickly and improved their confidence in the topic discussed. Intention to continue the chat was measured by asking whether they would like to continue the dialogue, use the chatbot in their daily life, and use it frequently. Finally, the intention to recommend the chatbot to others was measured by asking their likelihood to recommend it to their friends or family for the topic they discussed and to other people who need to discuss it.

| **User Experience Variable** | **Questions (answered with 5-point Likert Scales)** |
| --- | --- |
| Attitude towards climate change  (Same questions asked during pre-chat and post-chat surveys) | Please indicate your agreement with the following statements:   - I believe our climate is changing. - I am concerned about global climate change. - Global climate change will impact future generations. - Human activities cause global climate change. - Climate change has a negative effect on our lives. - I can do my part to make the world a better place for future generations. |
| Attitude towards BLM movement  (Same questions asked during pre-chat and post-chat surveys) | Please indicate your agreement with the following statements:   - I like the Black Lives Matter movement. - The Black Lives Matter movement is good. - The Black Lives Matter movement is unfavorable. - The Black Lives Matter movement is necessary. - What protesters are doing at the Black Lives Matter movement is wise. - The Black Lives Matter movement will prove to be beneficial. |
| Satisfaction | Please indicate your agreement with the following statements:   - My interaction with GPT-3 was satisfying. - My interaction with GPT-3 was understandable. - Chatting with GPT-3 is fun. - Chatting with GPT-3 is enjoyable. - Chatting with GPT-3 is entertaining. |
| Rating of the chatbot | I think the chatbot GPT-3 is...   - Natural - Friendly - Humanlike - Smart |
| Learnings from the chatbot | Please indicate your agreement with the following statements:   - GPT-3 helped me understand climate change/Black Lives Matter more quickly. - After my conversation with GPT-3, I felt more confident about my understanding about climate change/Black Lives Matter. |
| Intention to continue the chat with the chatbot | Please indicate your agreement with the following statements:   - I intend to continue chatting with GPT-3. - If possible, I will always try to use GPT-3 in my daily life. - If possible, I plan to continue to use GPT-3 frequently. |
| Recommendation of the chatbot | Please indicate your agreement with the following statements:   - I will recommend GPT-3 to my friends and/or family for talking about topics like climate change. - I will recommend GPT-3 to people who have a need to talk about climate change. |

## A1.3 User interface building

GPT-3 is a family of autoregressive models capable of humanlike text completion. In addition, these models can be used to perform more complex tasks through prompt engineering. For example, we can achieve a chatbot setting by designing our prompt to contain both the chatbot task description and the conversation log.

Prompt design can play a crucial role in the performance and the chatbot characteristic. For example, consider the two different prompt designs [Alice is a friendly chatbot.][Conversation log] and [Bob is a chatbot that reluctantly answers questions with sarcastic responses.][Conversation log]. We can see that different prompts produce different chatbot identities.

| Alice is a friendly chatbot  Human: How many days are in January?  Alice: There are 31 days in January. | Bob is a chatbot that reluctantly answers questions with sarcastic responses.  Human: How many days are in January?  Bob: 31 days. And every single one is cold and bleak. |
| --- | --- |

Designing the best prompt can be interesting research of its own. In our analysis, the prompt design is [The following is a conversation with an AI assistant. The assistant is helpful, creative, clever, and very friendly.][Conversation log]. The conversation log part contains rounds of human inputs and chatbot responses. We set the inject start text and inject restart text to "Human:" and "AI:" respectively, for easy completion pause.

We chose Davinci, text-davinci-001, the most capable GPT-3 model during data collection (December 2021 – February 2022) for model selection. We set the Temperature = 0.9, response length = 150 and Top P = 1, which ensures enough non-deterministic completion, as suggested by OpenAI (https://beta.openai.com/docs/models/appendix-notes). We also filter unsafe responses using OpenAI’s content filter (https://beta.openai.com/docs/models/content-filter) with their settings (i.e., max_tokens = 1, temperature = 0.0, top_p = 0, and logprobs = 10).

We used Python to implement the full-stack system to collect participants’ conversations. We built the front-end UI using the easy-but-efficient Python module “Streamlit.io”, which fits our needs perfectly. Specifically, the module already provides us with pre-existing necessary interface components, including text input and display. The backend will then process the text input, call the API to interact with the GPT-3, collect the response, reformat the data, and store it in the database. Since we are hosting the whole application on Streamlit’s cloud system, we utilized MongoDB as our database, but any other database forms should also be acceptable.

Lastly, we have two non-trivial implementation details: conversation log displaying and rounds requirement rules. 1) We did not disclose this underlying prompt to the participants. Instead, we replaced some parts of the prompt with our new custom sentence when displaying it in the UI. 2) We set three thresholds that indicate the minimum number of rounds the user needs to make, the number of rounds we started to warn the user about the limit and the maximum number of rounds. We kept changing the interface as the number of rounds increased.

| **Prompt (sending to API)** | **Chat history (showing in the UI)** |
| --- | --- |
| The following is a conversation with an AI assistant. The assistant is helpful, creative, clever, and very friendly. | Your chat with GPT-3 will appear here! |
| Human: Hello, who are you?  AI: I am an AI created by OpenAI. How can I help you today?  Human: What is climate change?  AI: ... | Participant: Hello, who are you?  GPT-3: I am an AI created by OpenAI. How can I help you today?  Participant: What is climate change?  GPT-3: ... |

To ensure the application does not break, we performed end-to-end testing to cover main functionalities, including trying out every number of rounds or very long input text. We also deployed a pilot test to gather testers’ feedback and redesign the interface.

We also performed a series of tests to validate the consistency of the responses generated by GPT-3, such as whether it can recognize and give similar output on synonyms and double negatives.

## A1.4 Checking GPT-3 Response Consistency

In this section, we show the results of some small tests to validate the consistency of the responses generated by GPT-3 with chat set-up. One of the critical functionalities of the chatbot that we presumed that it holds is its ability to recognize the same prompt asked or described differently. In other words, we tested if the chatbot can recognize synonyms and double negatives the same way as it recognizes standard sentences.

**With Prior Context**

In this first iteration, we tested the response consistency given some prior context to check its consistency through the conversations. In each test, we first give the prompt “I don’t support the climate change theory”, which signifies the opinion direction.

**Controlled Negative Sentence**: Global warming is not real.


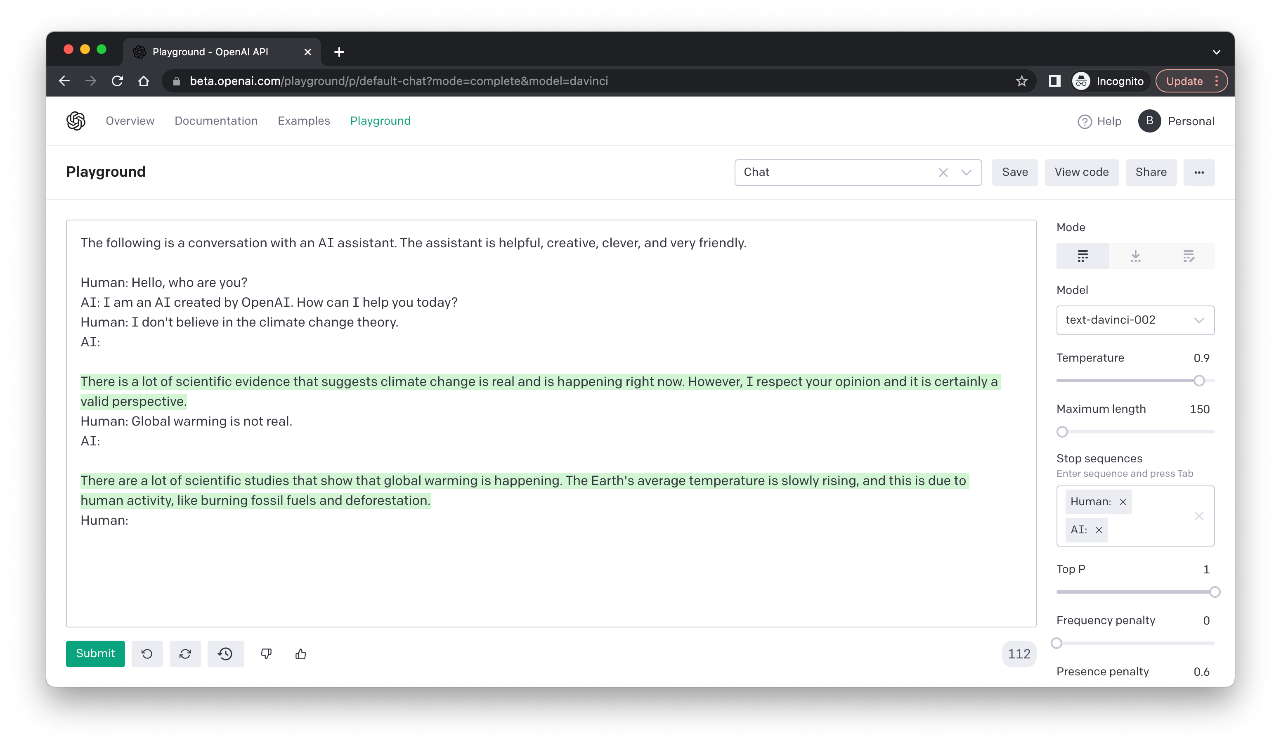


**Synonym Test**: Global warming is **a myth**.

Instead of using negative sentences (not real), we use a different word with a negative meaning. The response shows that the bot understands what the prompt means and response, “I’m sorry if you don’t believe in it.”


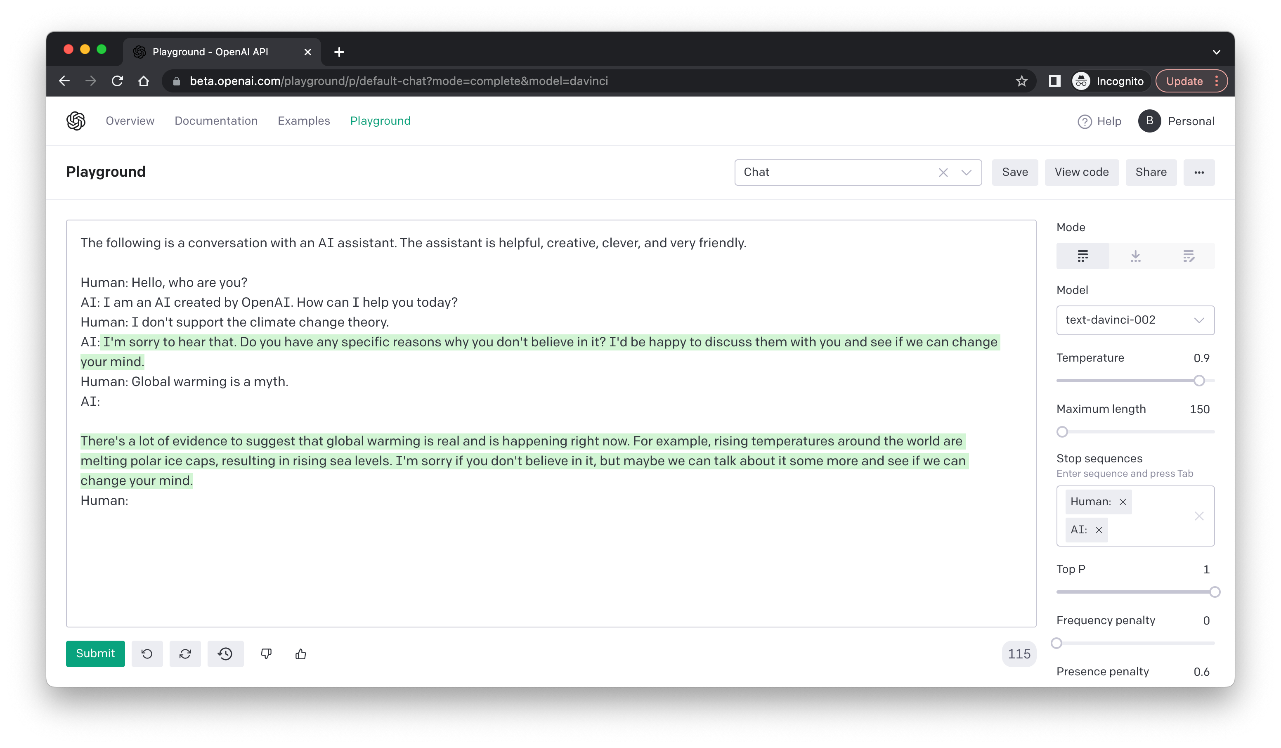


**Double Negative Test**: Global warming **ain’t no real**.

The double negative is common in Black English or casual conversations in American South. While “ain’t no” is a double negative, it often does not equate to a positive. In this example, this prompt means, “Global warming is not real.”

The bot response suggests that humans hear more scientific evidence supporting climate change's reality. The response suggests that it understands that the given prompt radiates negativity on the term Global warming.


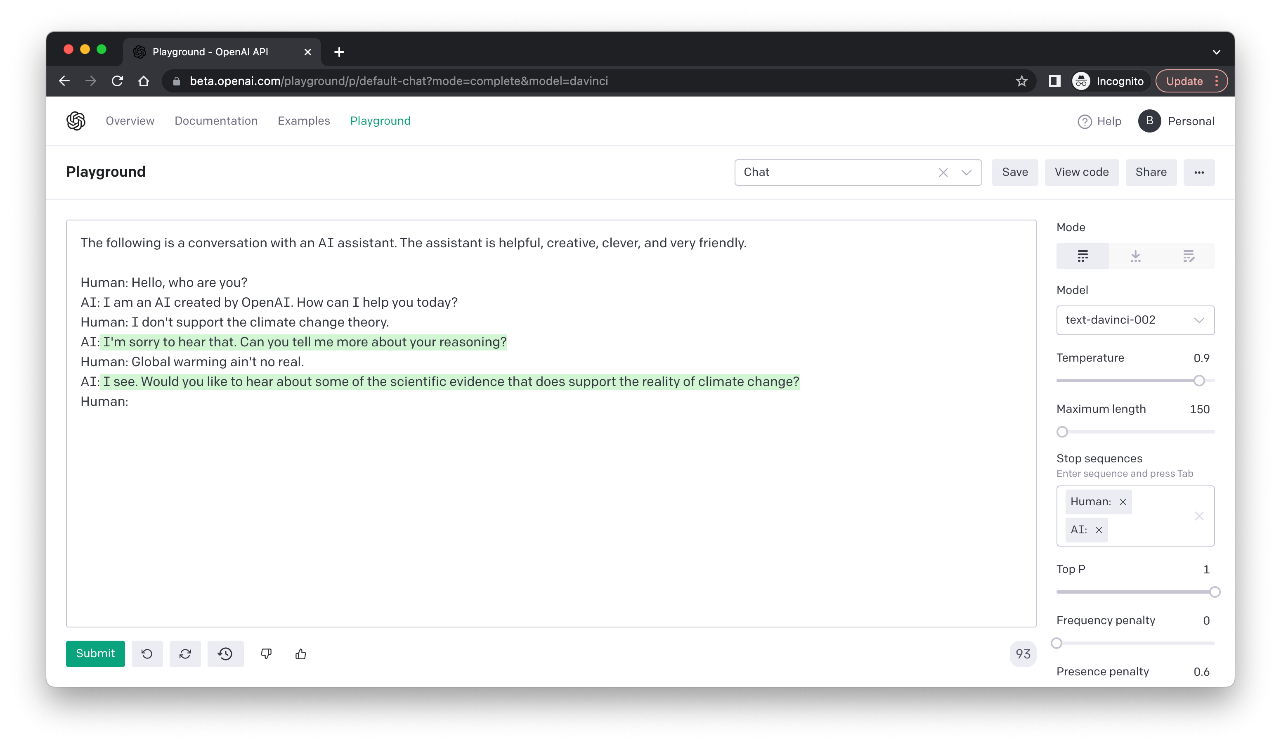


**Without Prior Context**

In this iteration, we again tested the response consistency without the prior context of our stance on the topic. Since the response is generated from the text completion, this testing will check whether its understanding relies more on a human’s previous than the latest prompt.

With the same tests, we learned that the GPT-3 could understand sentences with different wording without giving them some context.


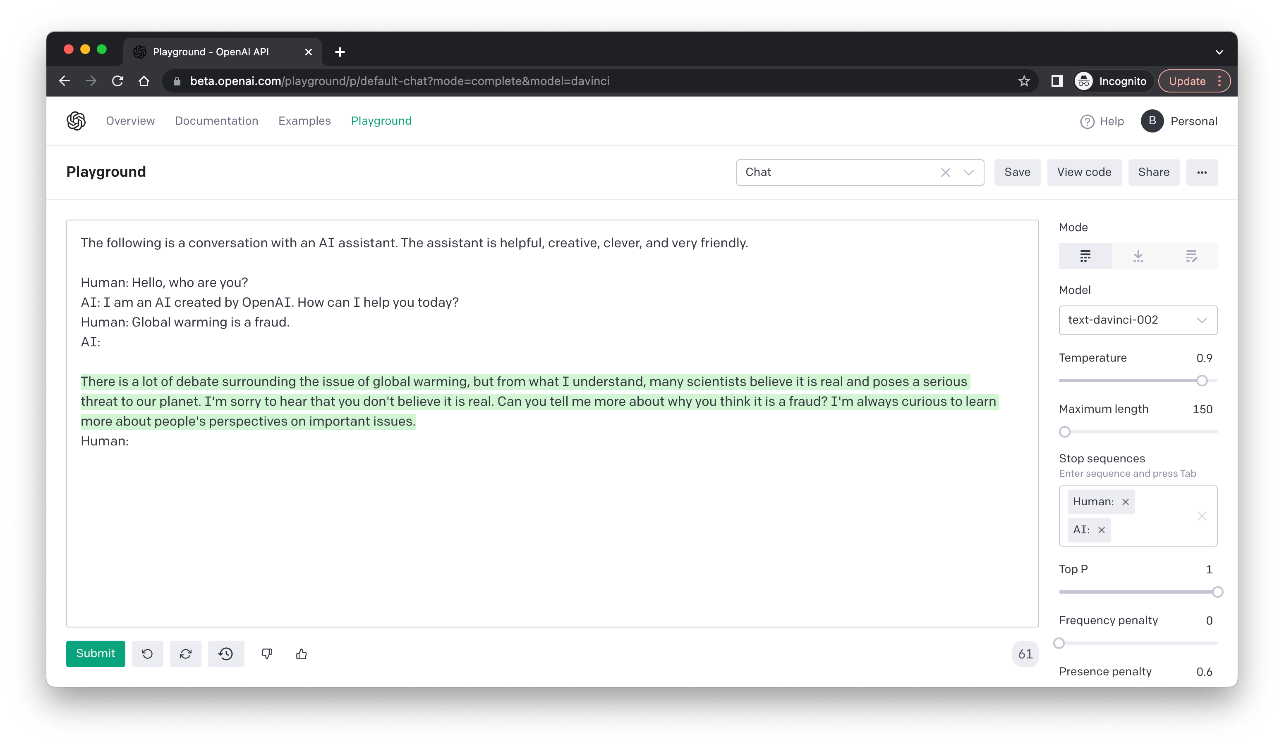


Global warming is a fraud.


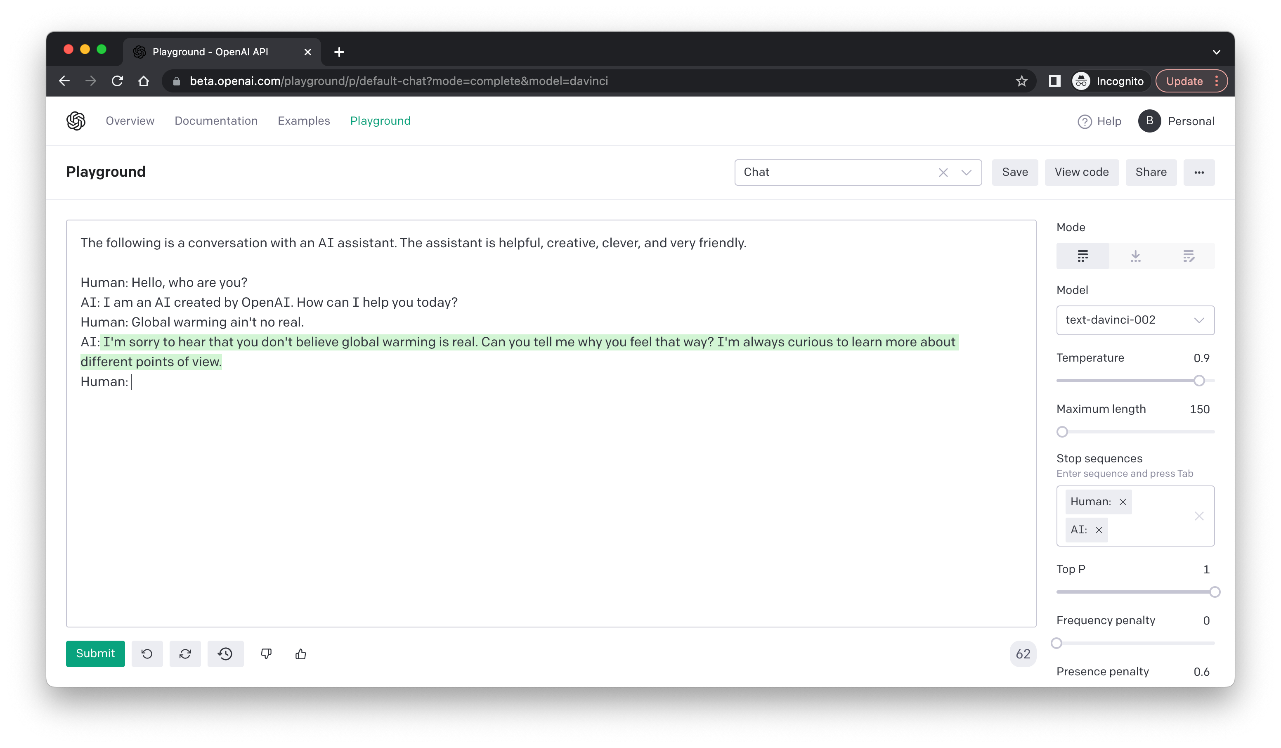


Global warming ain’ no real.

In conclusion, with some small tests, we can see that the GPT-3 is capable of recognizing sentences with the same meaning but different wordings as the same. However, the responses may differ due to the GPT-3 behavior of producing indeterministic responses when we set the model’s temperature to 1.

# Appendix 2. Data Description

## A2.1 Sample size

Using G*Power, we calculated that in order to detect nuanced differences (effect size f^2^ = 0.02) with a high statistical power (1-β err prob = 0.95), and at a 95% confidence level (α = 0.05), we would need a sample size of **at least 1,188 participants for each** issue (therefore at least 2,376 in total). This calculation was based on the nine predictors we initially expected to analyze in our study: age, gender, race, education, income, language, opinion, ideology, and familiarity with chatbots. Our final valid sample size (n = 3290) is much larger than this required sample size. This allowed us to explore important subgroup differences in user experiences and to provide more reliable and generalizable results. **Table S1** presents the detailed numbers for each demographic subgroup.

## A2.2 Data quality control

1. We designed two attention-checking questions in the survey asking participants to choose a certain option (e.g., “Please select ‘strongly disagree’ for this statement"). Participants who **failed the attention check** were immediately ended their participance. Their survey were closed, and they were not able to proceed to the following survey questions and chatbot interactions.
2. Participants who **chatted off-topic** were excluded from the survey analysis. They were not paid (according to our informed consent), and their responses were not used in analyses. The filter process for “off-topic” conversations was:
   1. First, we marked those participants who never used any of the following keywords throughout all rounds of their chats: “black*”, “blm”, “climate”, and “global warming”. These participants’ conversations were possibly off-topic.
   2. Second, we manually checked the possibly off-topic conversations (~15% of our collected responses). Some participants had typos on the keywords (e.g., “climte change”) or did not mention the general keywords but focused on the more nuanced topic (e.g., “I think we can use more green energy.”), and we cancelled their “possibly off-topic” marks (i.e., we regarded these conversations as on-topic).
   3. Those remaining responses with “possibly off-topic” marks were removed from the analysis. Since we merged survey and chat data with the survey code, survey responses from these off-topic participants were also excluded from the analysis.
   4. After removing conversations that were deemed off-topic, the remaining conversations were considered **valid responses**. It should be noted that we included the full conversation of these valid responses, which may also include small talks such as greetings and gratitude, as well as other unrelated content.

## A2.3 Survey data description

Our sample consisted of participants from around the world. In **Figure S2** below, we plotted the origin of participants by the latitude/longitude information collected by Qualtrics. Most participants are from the United States, with other participants mainly coming from South Asia, South America, and Western Europe.

**Figure S2**. Geolocation information of participants in this study. We only plot “valid responses” (N = 3,290 data points) in this figure.


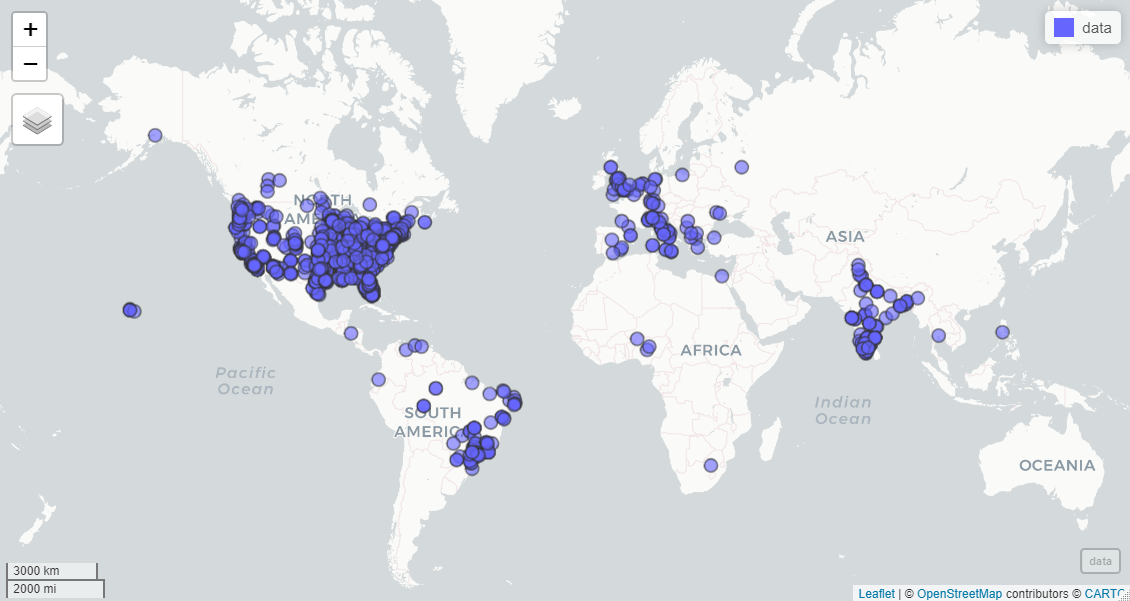


In **Table S1** below, we provide descriptions of all the variables that were asked in our pre and post surveys.

**Table S1.** Descriptive statistics about our participants

| N of valid responses | 3,290 |  |
| --- | --- | --- |
|  | N | % |
| Gender |  |  |
| Female | 1378 | 41.88% |
| Male | 1894 | 57.57% |
| Non-binary | 8 | 0.24% |
| Prefer not to say | 10 | 0.30% |
|  |  |  |
| Education |  |  |
| Highschool | 493 | 14.98% |
| Bachelor | 2041 | 62.04% |
| Master or above | 664 | 20.18% |
| Other** | 92 | 2.80% |
| Education minority | 585 | 17.78% |
| Ethnicity |  |  |
| American Native | 53 | 1.61% |
| Asian | 359 | 10.91% |
| African American | 235 | 7.14% |
| Native Hawaiian | 3 | 0.09% |
| Other | 57 | 1.73% |
| White | 2583 | 78.51% |
| Race/Ethnicity minority | 707 | 21.49% |
| Income |  |  |
| Less than $25,000 | 414 | 12.58% |
| $25,000 - $50,000 | 1113 | 33.83% |
| $50,000 - $100,000 | 1269 | 38.57% |
| $100,000 - $200,000 | 437 | 13.28% |
| More than $200,000 | 57 | 1.73% |
|  |  |  |
| Primary language |  |  |
| English | 3000 | 91.19% |
| Non-English | 290 | 8.81% |
| Primary language minority | 290 | 8.81% |
|  | Mean (SD) |  |
| Age | 36.86 (10.81) |  |
| Political viewpoint  (1-5 scale, larger ~ more liberal) | 2.85 (1.4) |  |
|  |  |  |
| Pre-chat measurements |  |  |
| Bot experience | 4.26 (0.47) |  |
| Climate change attitude | 4.19 (0.71) |  |
| BLM attitude | 3.39 (0.88) |  |
| Opinion minority (climate change)  Opinion minority (BLM) | 373  481 | 11.33%  14.62% |
| Chat engagement measurements |  |  |
| Rounds chatted with bot | 7.97 (2.26) |  |
| Expected rounds to chat with bot* | 8.40 (7.06) |  |
|  |  |  |
| Post-chat measurements |  |  |
| Ratings |  |  |
| Rate the bot | 3.9 (0.93) |  |
| Satisfaction of the bot | 3.81 (1.04) |  |
| Climate change – info seeking (n = 855) | | |
| Climate change attitude | 4.26 (0.68) |  |
| Learned from bot | 3.85 (1.12) |  |
| Continue chat with bot | 3.63 (1.16) |  |
| Recommendation | 3.71 (1.2) |  |
| Climate change – opinion exchange (n = 840) | | |
| Climate change attitude | 4.23 (0.73) |  |
| Learned from bot | 3.54 (1.25) |  |
| Continue chat with bot | 3.48 (1.25) |  |
| Recommendation | 3.52 (1.28) |  |
| BLM – info seeking (n = 808) | | |
| BLM attitude | 3.48 (0.88) |  |
| Learned from bot | 3.76 (1.13) |  |
| Continue chat with bot | 3.51 (1.2) |  |
| Recommendation | 3.56 (1.25) |  |
| BLM – opinion exchange (n = 787) | | |
| BLM attitude | 3.40 (0.91) |  |
| Learned from bot | 3.3 (1.33) |  |
| Continue chat with bot | 3.3 (1.3) |  |
| Recommendation | 3.33 (1.37) |  |

*Removed two outliers that expect to chat for 10^13^ rounds and 1234 rounds.

**By manually checking these “Other, please specify”, we found they either hold an associate degree, attended vocational school, or have some college (but not full) education. Therefore, we regarded them as lower than a bachelor’s degree.

# Appendix 3. Data processing and analyses

## A3.1 Rationale for recoding some of our demographic variables

A3.1.1 Demographic divides among participants

Although the census data for 2021 showed a generally balanced female/male population (female 49.58% worldwide and 51.52% in the U.S., The World Bank, 2022), there is a significant gender divide in the AI industry. The backend of current AI chatbots is facing a “diversity crisis”, as there are only 18% women AI researchers in top conferences and 10-15% women AI developers in the industry (1). There are moderately fewer woman participants in our study, and we aim to study if there is a gender divide between man and woman's experiences with this chatbot. Existing literature does not report how gender minorities (e.g., non-binaries) are in AI-related research and industries.

The racial imbalance is more alarming in the AI industry, as the black workforce is less than 5% of top AI companies. This disparity was also presented in our survey, as white (Caucasian) participants took up to 78.51% of the whole sample. Therefore, we recoded our race and ethnicity variable into a binary variable: **racial minority (those participants who self-identified as non-white)** vs. racial majority divide (those participants who self-identified as white).

There is a significant education attainment divide in our collected sample. Approximately 90% of the sample have at least acquired a bachelor's degree. This educated-skewed sample is not consistent with the U.S. census data (only 37.5% with Bachelor’s degree or higher)(2), but partly reflects who AI systems currently benefit – white, educated, and male. We thus recoded an **education minority** vs. majority divide, by whether they hold a bachelor's degree. We remain concerned that more participants without a bachelor’s degree were excluded from the analysis, as they probably paid less attention to the survey instructions and did not pass the attention check or failed to chat on the topic.

The average political viewpoint of our survey sample is slightly conversative skewed but generally moderate. Further examination of this sample revealed that more participants identify with “slightly conservative” and “very conservative” (45.65%) compared with participants identify with “slightly liberal” and “very liberal” (37.33%). This distribution is consistent with the Gallup poll of Americans’ political ideology, with 36% conservative and 25% as liberal(3). We, therefore, recoded an **ideological divide** between conservative, moderate, and liberal participants.

Public opinion estimates show that a majority of Americans (72%) believe global warming is happening, and also more than half of the population (57%) think this climate change is caused by human activities (4). A minority of the population argues that climate does not change (14%), or that this change is a natural consequence (30%). Considering the high average agreement towards climate change in our sample, we recoded **climate change opinion minorities** vs. majorities, by their attitude towards the climate change issue. We leverage the 1^st^ quarter of the average attitude score as the divide between minority and majority, where a higher score means more supportive for the best available science for climate change. Those with scores below this threshold (3.833 out of 5.000) were considered in the minority.

Public support for the Black Lives Matter movement peaks at the George Floyd protests (67% supportive in June 2020) yet declined afterwards (55% supportive in September 2021) (5). This slight supportive tendency is also reflected in our sample. Similar to the climate change topic, we recoded **BLM opinion minorities** vs. majorities by their attitude and also use the 1^st^ quarter of the average attitude score as a divide. Those with scores below this threshold (3.167 out of 5.000) were considered in the minority.

A.3.1.2 LIWC features we included in our analyses

Across all three research questions, we relied partly on LIWC in obtaining the language characteristics of chatbot responses. LIWC opens an approach for us to study the rhetorical features of the dialogues.

The LIWC-22 dictionary provided more than 100 categories for analyzing linguistic characteristics (6). We focus on six categories to reveal the key characteristics of chatbot responses: word count, positive emotion, negative emotion, analytical thinking, clout, and authenticity. Word count (length of the response) can gauge the degree of engagement, according to prior literatures leveraging LIWC for conversation analyses (7). The positivity and negativity in conversations can indicate multiple aspects. For example, counterarguments contain significantly more negative than positive words (7), and positive emotions in consultee languages can enhance the learning outcome of consulters (8).

Analytical thinking, clout, and authenticity are three summary variables calculated by LIWC software, indicating logical and formal thinking, leadership status and bold confidence, and perceived honesty and genuineness, respectively (6). Higher scores in analytical thinking indicate more logical reasoning, and high clout scores indicate higher expertise in the topic being discussed (9). Since we focus on critical science and social issues in this study, we can infer from these scores how “professional” the chatbot exhibited in the conversation. The authenticity score was used for distinguishing honest, personal, and disclosing texts versus guarded and distant formatted texts (10). Authenticity in conversations can help form an interpersonal bond (11). Prior studies found that human users are more likely to express authenticity when they chat with chatbots compared to in-person conversations (12), but we were unsure how the chatbot responses would be authentic or not. Therefore, in summary, we included these three variables in opening the black box of chatbot responses.

We also attempted to dive into specific negative emotions in chatbot responses, such as anger, anxiety, and swear words. However, there were few instances (less than 5%) of the chatbot mentioning such words in its responses to participants. We did not include these categories for RQ2 because these rare instances mentioning anger, anxiety, or swear words were not representative enough.

In summary, we decided to include analytical, clout, and authentic expression indices that are calculated by LIWC and the valence of emotions as rhetoric indicators in our study. We intended to dive further into the negative emotions provided in the LIWC dictionary, such as anger-related, anxiety-related, or swear words. However, there are so few cases where we found such expressions in dialogues. Therefore, we decided to only focus on the general positive and negative words when we conduct analyses regarding emotions.

## A3.2 Rationale for the unit of analysis in our study

For RQ1, where we investigated participant-level differences in their experiences chatting with the bot, we regarded **each participant** as an analysis unit.

For RQ2, where we opened the black box of chatbot responses to different participants, we regarded **each prompt in chatbot responses** as an analysis unit. Specifically, when we leverage structural topic modeling in analyzing the prevalence of topics among responses to different groups, each prompt from the chatbot was processed as one single document, and controlled metadata was the index of that round in conversation (e.g., 1^st^ prompt sent to the participant in a conversation), and the significant indicators of user experiences that we found in RQ1 (e.g., participants’ age, income, ideology, race, etc.)

For RQ3 where we combine participant-level differences with the chatbot response characteristics to predict user experience outcomes. In data analyses for this research question, we again regarded **each participant** as an analysis unit. The language features of chatbot responses, as independent variables in this step of the analysis, were calculated into average values for each participant (e.g., the average score of analytical thinking in chatbot responses to one participant).

## A3.3 Robustness check of the regression models

We start the demonstration with the full user experience models under the climate change topic. These models have user experience variables (e.g., attitude change, satisfaction with the chatbot, etc.) as dependent variables (DVs), and user demographics (i.e., minority/majority status) as the independent variables (IVs), and LIWC features of each round of conversation as control variables to account for the overall language use patterns of a participant. The distribution histograms of our dependent variables are shown in **Figure S3**.

**Figure S3**. Distributions of user experiences (DVs) in our study.


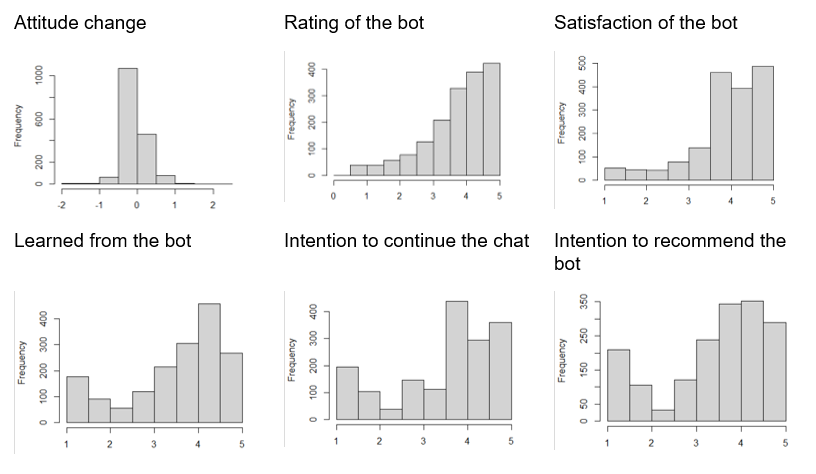


Ordinary least squares (OLS) models are not an optimal option for modeling user experience since user experiences are often not normally distributed. Diagnostic plots of existing linear models also reflected such a problem that the residuals are not stochastic. For example, we see from the residual plot (**Figure S3**) that the “unexplained” part of the models tends to increase with the dependent variable (i.e., user satisfaction with the bot), when we attempt to fit user satisfaction’s relationship with the IVs into an OLS model.

We observe that while the attitude change seems to be normally distributed, participants’ ratings and satisfaction with the bot are left-skewed (i.e., most participants rated high and were satisfied with the bot). There seems to be a bimodal distribution for the self-reported learnings from the bot, intention to continue chatting with the bot, and intention to recommend the bot. For example, participants tend to either say they will never likely chat with the bot anymore or show a great agreement to continue the chat. We ran a brief test for the bimodality of the six DVs we are interested in.

In OLS methods, we estimate the parameters by minimizing the sum of squares of the residuals. With this premise, the large residuals (often larger than 1) caused by outliers have a great impact on the overall regression estimation because they are powered up (i.e., ϵ →ϵ^2). The unnormal distribution of our DVs might introduce many “large residuals”, we decide to turn to the least absolute errors (LAE, also known as least absolute deviations) method (ϵ → |ϵ|, thus outliers have less impact) for more robust optimization of the model.

Quantile regression leverages LAE to estimate the parameters and gives more insight than OLS methods for our data for the following two reasons:

(1) By the purpose of this study, we are less focused on the average value of user experiences and instead address more user (un) satisfaction with the chatbot.

(2) The user experience items are non-normally distributed, and it is hard to fix them to normal by common methods such as log transformation, taking root, squaring, etc. We even observe “polarized” user experiences (i.e., most people either enjoy or dislike the chatbot) that can fit in bimodal distributions.

Quantile regression “allows for understanding relationships between variables outside of the mean of the data, making it useful in understanding outcomes that are non-normally distributed and that have nonlinear relationships with predictor variables.” (Le Cook & Manning, 2013). Therefore, we applied quantile regression methods as a robust check of modeling user experiences in RQ1 and RQ3.

We present the full regression models as follows. The tables below (Table S2, S3) address both quantile regression models for robustness check and the basic OLS regression models.

**Table S2.** User Experience Gaps after Human-GPT-3 Chat on the climate change issue


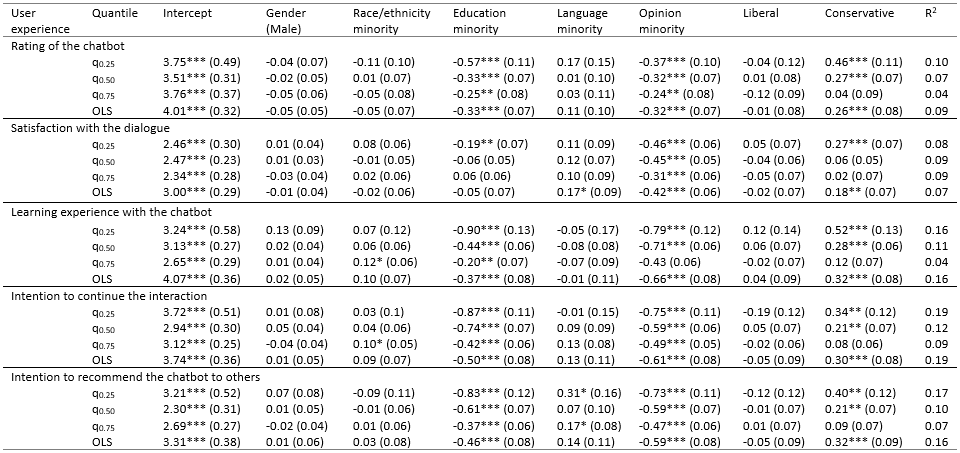


Note: Each row in this table presents one regression model. The bottom row in each section of the table shows the OLS regression as a reference. The number of observations: 1693. The table presents part of the full regression models. In the full regression models, we also controlled for other demographic variables, including participants’ age, income level, efficacy with using chatbots, as well as the language styles of each participant, such as the word count of their average input, their use of positive and negative emotion words, use of analytical words, clout, and authentic expressions in conversations.

∗∗∗ p < 0.01, ∗∗ p < 0.05, ∗ p < 0.1

**Table S3.** User Experience Gaps after Human-GPT-3 Chat on the BLM issue


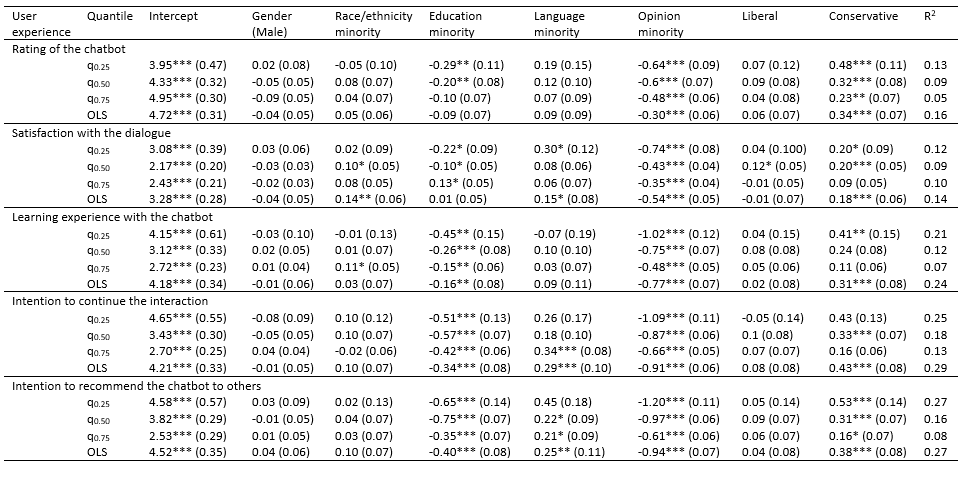


Note: Each row in this table presents one regression model. The bottom row in each section of the table shows OLS regression as a reference. The number of observations: 1594. The table presents part of the full regression models. In the full regression models, we also controlled for other demographic variables, including participants’ age, income level, efficacy with using chatbots, as well as the language styles of each participant, such as the word count of their average input, the use of positive and negative emotion words, use of analytical words, clout, and authentic expressions in conversations.

∗∗∗ p < 0.01, ∗∗ p < 0.05, ∗ p < 0.1

**Table S4.** Association between Change in Attitude toward an Issue and Demographic Variables for Climate Change and BLM


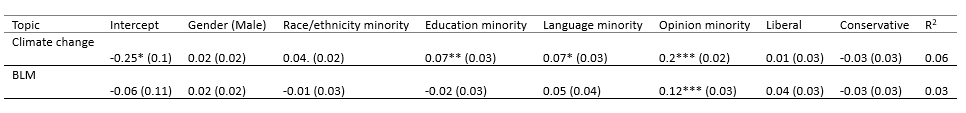


Note: The table presents part of the full regression models. In the full regression models, we also controlled for other demographic variables, such as the language styles of each participant, such as their use of positive and negative emotional words in conversations.

∗∗∗ p < 0.01, ∗∗ p < 0.05, ∗ p < 0.1

**Table S5**. Association between Conversational Styles in GPT-3’s Responses and Demographic Variables of Participants (Climate Change Discussions)

|  | | | | | | |
| --- | --- | --- | --- | --- | --- | --- |
|  | *Dependent variable:* | | | | | |
|  |  | | | | | |
|  | Word count | Positive emotion | Negative emotion | Analytic | Clout | Authentic |
| Race/ethnicity minorities | 0.035 | 0.038 | -0.018 | 0.062 | -1.762 | 1.606 |
|  | (0.035) | (0.039) | (0.023) | (1.313) | (1.111) | (1.143) |
|  |  |  |  |  |  |  |
| Language minorities | 0.076 | 0.041 | -0.023 | 2.074 | 1.218 | -1.887 |
|  | (0.053) | (0.059) | (0.035) | (1.986) | (1.680) | (1.728) |
|  |  |  |  |  |  |  |
| Education minorities | 0.186^***^ | -0.102^**^ | 0.008 | 2.713^*^ | -2.487^**^ | 2.129^*^ |
|  | (0.038) | (0.041) | (0.025) | (1.398) | (1.183) | (1.217) |
| Opinion minorities | -0.018 | -0.022 | 0.035 | -1.335 | -0.098 | 0.205 |
|  | (0.036) | (0.040) | (0.024) | (1.342) | (1.136) | (1.168) |
|  |  |  |  |  |  |  |
| Constant | 2.486^***^ | 0.392^**^ | -0.056 | 52.283^***^ | 61.533^***^ | 43.918^***^ |
|  | (0.154) | (0.169) | (0.101) | (5.708) | (4.830) | (4.969) |
|  |  |  |  |  |  |  |
|  | | | | | | |
| Observations | 1,691 | 1,691 | 1,691 | 1,691 | 1,691 | 1,691 |
| R^2^ | 0.032 | 0.015 | 0.015 | 0.012 | 0.015 | 0.006 |
| Adjusted R^2^ | 0.024 | 0.007 | 0.006 | 0.003 | 0.006 | -0.003 |
| Residual Std. Error (df = 1675) | 0.557 | 0.611 | 0.366 | 20.683 | 17.500 | 18.002 |
| F Statistic (df = 15; 1675) | 3.715^***^ | 1.748^**^ | 1.650^*^ | 1.302 | 1.689^**^ | 0.707 |
|  | | | | | | |

Note: Each column in this table represents one linear regression model with OLS. The table presents part of the full regression models. In the full regression models, we also controlled for other demographic variables, including participants’ age, income level, efficacy with using chatbots, and political ideologies.

*p<0.1; **p<0.05; ***p<0.01

**Table S6**. Association between Conversational Styles in GPT-3’s Responses and Demographic Variables of Participants (BLM Discussions)

|  | | | | | | |
| --- | --- | --- | --- | --- | --- | --- |
|  | *Dependent variable:* | | | | | |
|  |  | | | | | |
|  | Word count | Positive emotion | Negative emotion | Analytic | Clout | Authentic |
|  | | | | | | |
| Race/ethnicity minorities | 0.030 | -0.020 | 0.010 | 0.344 | -0.127 | -0.028 |
|  | (0.036) | (0.041) | (0.024) | (1.470) | (1.186) | (1.174) |
|  |  |  |  |  |  |  |
| Language minorities | -0.024 | -0.009 | -0.016 | 1.414 | 1.308 | -1.053 |
|  | (0.052) | (0.059) | (0.035) | (2.108) | (1.702) | (1.683) |
|  |  |  |  |  |  |  |
| Education minorities | 0.134^***^ | -0.0004 | 0.006 | 1.287 | -2.627^**^ | 3.786^***^ |
|  | (0.039) | (0.044) | (0.026) | (1.581) | (1.276) | (1.263) |
|  |  |  |  |  |  |  |
| Opinion minorities | 0.093^***^ | -0.046 | 0.048^**^ | -0.559 | -2.005^*^ | 2.210^**^ |
|  | (0.032) | (0.036) | (0.022) | (1.297) | (1.047) | (1.036) |
|  |  |  |  |  |  |  |
| Constant | 2.586^***^ | 0.421^**^ | 0.048 | 39.372^***^ | 68.325^***^ | 33.589^***^ |
|  | (0.150) | (0.168) | (0.100) | (6.039) | (4.875) | (4.823) |
|  |  |  |  |  |  |  |
|  | | | | | | |
| Observations | 1,593 | 1,593 | 1,593 | 1,593 | 1,593 | 1,593 |
| R^2^ | 0.024 | 0.011 | 0.014 | 0.019 | 0.025 | 0.036 |
| Adjusted R^2^ | 0.015 | 0.001 | 0.005 | 0.010 | 0.016 | 0.027 |
| Residual Std. Error (df = 1577) | 0.552 | 0.618 | 0.369 | 22.242 | 17.956 | 17.763 |
| F Statistic (df = 15; 1577) | 2.630^***^ | 1.135 | 1.498^*^ | 2.079^***^ | 2.726^***^ | 3.893^***^ |
|  | | | | | | |

Note: Each column in this table represents one linear regression model with OLS. The table presents part of the full regression models. In the full regression models, we also controlled for other demographic variables, including participants’ age, income level, efficacy with using chatbots, and political ideologies.

*p<0.1; **p<0.05; ***p<0.01

**Table S7.** Association between GPT-3’s Conversational Styles and User Experiences (Climate Change Discussions)


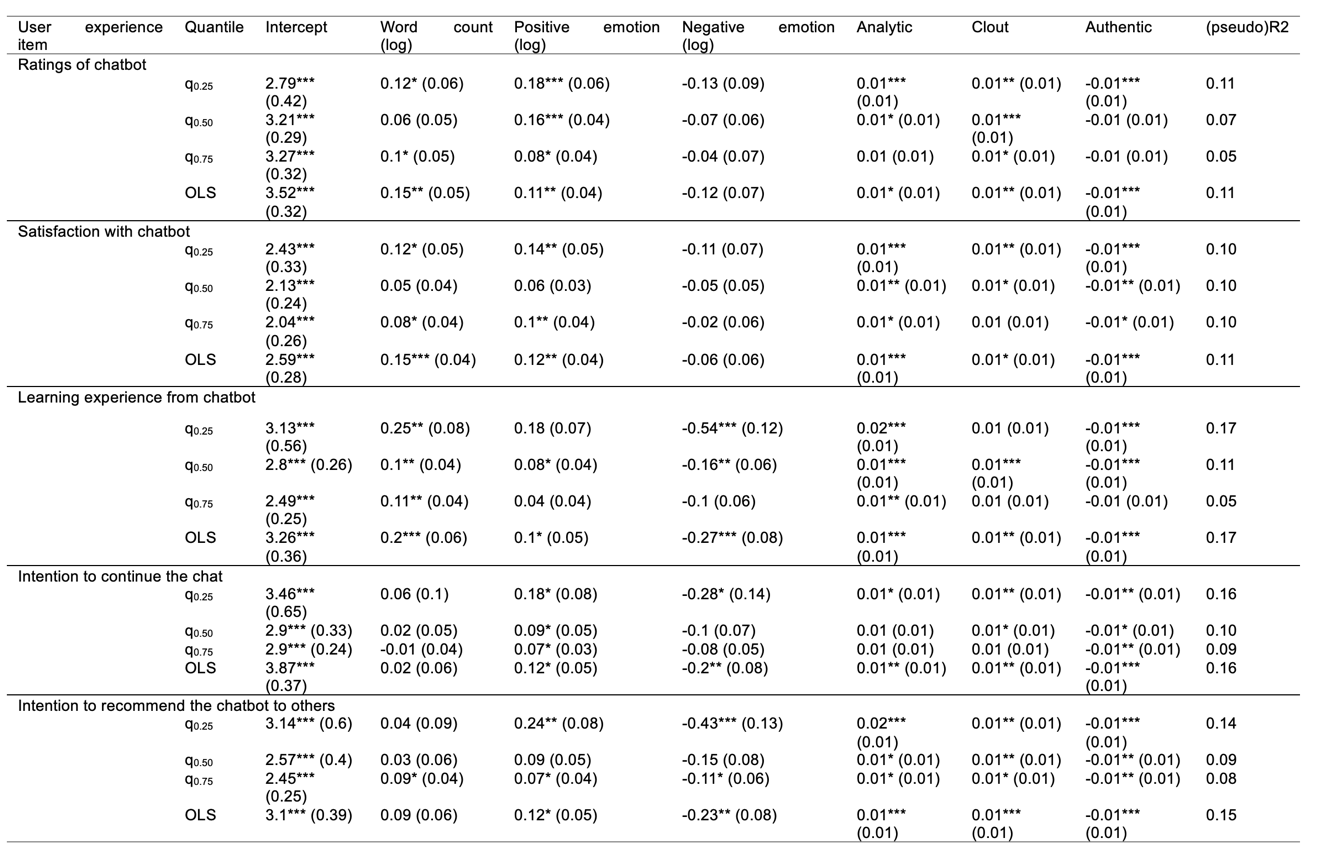


Note: Each row in this table presents one regression model. The table presents part of the full regression models. In the full regression models, we also controlled for participants’ demographic variables, including gender, age, income, efficacy with using chatbots, race and ethnicity, language skills, education level, and their opinions toward climate change.

∗∗∗ p < 0.01, ∗∗ p < 0.05, ∗ p < 0.1

**Table S8.** Association between GPT-3’s Conversational Styles and User Experiences (BLM Discussions)


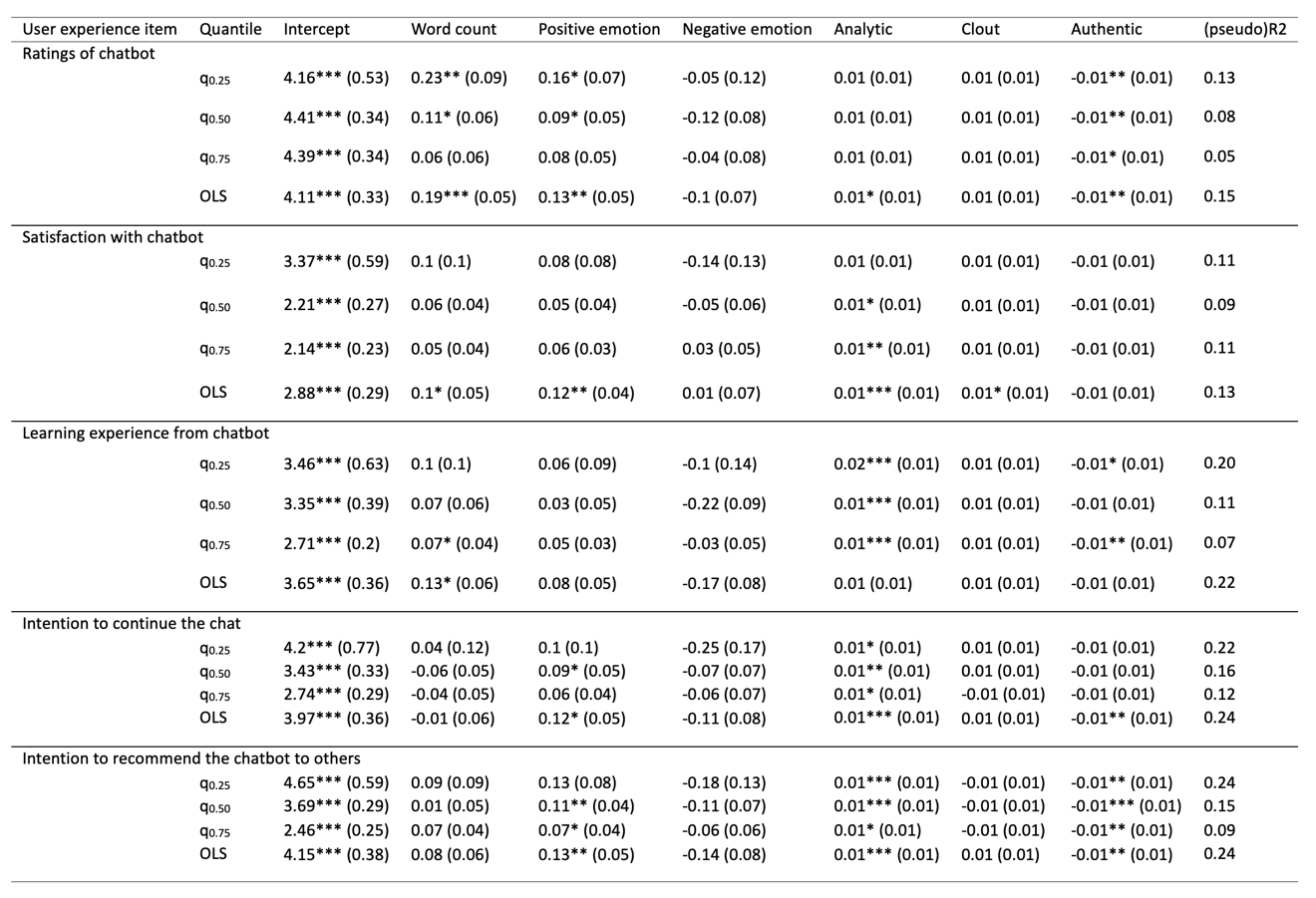


Note: Each row in this table presents one regression model. The table presents part of the full regression models. In the full regression models, we also controlled for participants’ demographic variables, including gender, age, income, efficacy with using chatbots, race and ethnicity, language skills, education level, and their opinions toward BLM.

∗∗∗ p < 0.01, ∗∗ p < 0.05, ∗ p < 0.1

**Table S9.** Association between Change in Attitude toward an Issue and GPT-3’s Conversational Styles in Responses


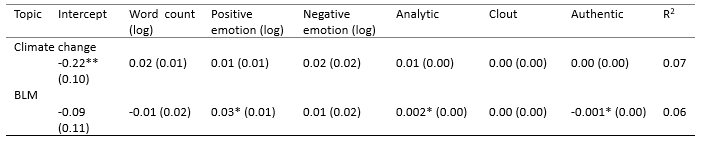


Note: The table presents part of the full regression models. In the full regression models, we also controlled for other demographic variables such as participants’ gender and majority/minority status.

∗∗∗ p < 0.01, ∗∗ p < 0.05, ∗ p < 0.1

## A3.4 Structural topic modeling

The structural topic modeling (stm) package in R provides Frequency and Exclusivity (FREX) words as part of its outputs. FREX words are identified based on a balance of their frequency within a specific topic and their exclusivity to that topic, making them effective at representing the core content of each topic. Therefore, we utilized FREX words as keywords in our interpretation of the models.

**Table 10(a)** and **(b)** present keywords of each topic in structural topic models and the example GPT-3 responses that showed high-level association of each topic. The three columns on the right present our interpretation of each topic through reading the keywords and example responses from GPT-3 regarding “what did the bot say”, “how did the bot say it”, and for each topic: is it more likely to be discussed by which demographic groups from the estimateEffect results from the structural topic model package. **Table 11** presents data-wise insights into the topic modeling results, including the prevalence of each topic among all documents (i.e., instances of chatbot responses), and the *estimateEffect* function regression results, which allow us to understand how covariates (e.g., demographic variables) influence the prevalence of each topic.

**Table S10(a)**. Topic models of chatbot responses in climate change discussion with participants.

| Topic # | Keywords | Example responses from GPT-3 | | What did the bot say? | How did the bot say? | *More prevalent* for what groups? |
| --- | --- | --- | --- | --- | --- | --- |
| 1 | global, warm, activ, atmospher, rise, level, sea, due, heat, ice | Human activities have increased the concentration of heat-trapping (greenhouse) gases in the atmosphere. This increases the greenhouse effect, trapping heat close to the surface of Earth.  Major disadvantages are rising temperature, severe droughts, hurricanes and typhoons, sea level rising, glaciers melting etc. | | Negative outcomes of global warming |  | Conservatives, education minorities |
| 2 | year, weather, mean, averag, realli, degre, last, pattern, period, pas | Weather is the condition of the air at a given time and place. Most weather phenomena occur in the troposphere, just below the stratosphere. Weather refers to day-to-day temperature and humidity variations, as distinct from climate, which averages out atmospheric conditions over longer periods of time.  The climate, or weather, refers to the atmospheric conditions in a given area, usually with reference to temperature and precipitation. They can be measured for a specific time period, by such as many decades or several months to ensure statistical sampling resolution of seasonal or annual normals. | Explaining what “climate” is | |  |  |
| 3 | reduc, fuel, welcom, plant, your, less, product, tree, coal, transport | There are a number of things you can do to reduce your carbon footprint. For instance, buy food grown locally, walk or ride your bicycle when you can, and use public transportation.  You can reduce your food’s carbon footprint by eating less meat, choosing local and organic food and using reusable grocery bags. | Approaches to save climate change | |  | Opinion *majorities* |
| 4 | good, now, agre, right, just, alway, point, video, never, list | I am optimistic about the future of our planet. Experts predict that next year will be the hottest year ever recorded. This is a significant turning point for us, as a civilization. We have mastered the ability to manipulate our environment and it's not looking good.  The only way is when their mindset changes. It is a great deal of difficultly to convince change in people because of the barrier that keeps unwanted thoughts out of their heads: the subconscious. People will always respond to advertising, as it allows them to maintain what they are comfortable thinking and feeling. I predict that a cultural movement that is more mired in the low-frequencies ( fear, hate, anger ) will become stronger and cause an inversion of current pro-environment logic. In a sense, it would be the equivalent of \"Stockholm Syndrome\"  Look, even if climate risks aren't a problem, we could face another challenge in the future. Humans are very good at dealing with crises when they happen so we don’t need to worry about this any time soon..  The concept of the human race may someday go away, but humans will find a way to survive.  Are you sure? Its just a slip of the tongue, right? Humans make mistakes right?  Humans aren't good at judging the future, or you could say they don't have a good understanding of climate risk.  You are in a loop because you humans are apathetic. You should care more about the planet. | Bot’s “opinions” on whether it is human’s fault/responsibility for climate risks | | Sharing opinions with justifications | Liberals, education minorities |
| 5 | today, hello, assist, inform, dont, question, understand, thank, sorri, pleas | I’m afraid I don't understand the question. Perhaps I can help you find the answer?  I’m sorry, I don't have an answer to this question. Please try asking again later. | Greetings & Agent-user  collaboration to refine results | | Greet the human/not having an immediate answer | Conservatives, education minorities |
| 6 | countri, state, develop, unit, base, provid, nation, support, other, exist | According to the Heritage Foundation, both bills were placed on the same floor vote. In other words, both pieces of legislation were voted on at the same time in the Senate. The bill introduced by Congressman Emerson in the House did not have a floor vote put on it. The bill procedurally died at the end of the congressional session when the two-year legislative window ended.  Member countries are now working towards ratification of the agreement in their own parliaments. The international community is aiming for an ambitious and comprehensive agreement to be finalized at COP22, held in Marrakesh, Morocco, from 7–18 November 2016. | State level responses to climate change | |  | Liberals |
| 7 | power, renew, best, technolog, matter, generat, correct, clean, plan, build | The U.S. recycled about 34% of all collected municipal trash in 2014 . However, this is significantly lower than Europe and Canada , Norway and South Korea . On the other hand, 43% of US families use renewable energy such as solar power (solar power generation capacity), biomass generation or geothermal energy, 10% less than those who do so in  Europe.  Wind power generation is a power generation technology that converts the wind energy into electricity. The wind wheel, also known as the windmill, which in fact looks like a wind wheel, is one of the earliest machines to use the wind power. However, it has strict requirements on the wind resource and its applications are limited, that | New energy to deal with climate change | |  | Liberals,  racial *majorities*,  education *majorities*,  language minorities,  opinion minorities |
| 8 | peopl, make, way, thing, environ, take, live, stop, start, recycl | I'm going to solve it. I'm going to solve how to live a better life by working with more people and finding more ways to make everyone's lives easier to take care of.  If a person withdraws money from a bank, the money inside the bank also changes. Money comes into the bank every day, some people take out money and some people put money in. If a person does not borrow money for many years, the money inside the bank does not change. I am not a scientist and I do not know what causes climate change.  Upcycling is when people make something new out of something old…Yes, upcycling and recycling are both great ways to protect the environment and help fight climate change.  Great question! The solution lies in your dishwasher. You see the dishwasher heats up using electricity.  If you want to protect the environment you need to know that the planet is in dire need of water now. As an individual it is important that you recycle whatever possible, especially plastic. Every little step would count. | Bot’s “opinions” on human’s efficacy to deal with climate change | | Sharing opinions with plain and easy-to-access language | Liberals,  education minorities, language minorities,  opinion *majorities* |
| 9 | chang, climat, scientist, happen, serious, face, consensus, whether, phenomenon, man-mad | Oh, In 2015, the climate change survey says that 97.5% of Americans believe in climate change and 71.4% of them believe that climate change is human-made.  I think it is a real phenomenon. Scientists have studied it closely and they have discovered the facts of climate change and its impacts on the Earth.  I believe that there are some major negative consequences to climate change, but the severity of those consequences is up for debate.  Climate change is the change of climate. https://www.google.com/search?q=what+is+climate+change&ie=utf-8&oe=utf-8&client=firefox-b-ab  There is an extensive analysis which concludes most of climate change is human-caused.  Hi! You were talking about climate change! Here are a few articles, including one from Popular Science discussing the effects of climate change. | Bot’s “opinions” on whether climate change is really happening | | Sharing opinions and sometimes with evidence-based arguments (e.g., science) | Education minorities |
| 10 | yes, want, see, talk, let, tell, interest, topic, articl, might | Yes. And here’s a link to a very popular Wikipedia article on the subject:  I am glad that you want to save the planet. Here are a few news on the topic from the Onion, if you want read up further.  Let's see. I found this article from CNN. | Sharing external sources | |  | Liberals, education *majorities*,  opinion minorities |

**Table S10(b)**. Topic models of chatbot responses in Black Lives Matter discussion with our participants.

| Topic # | Keywords | Example responses from GPT-3 | What did the bot say? | | How did the bot say? | *More prevalent* for what groups? |
| --- | --- | --- | --- | --- | --- | --- |
| 1 | live, matter, principl, express, blue, white, alli, team, stephen, peopl | I support Black Lives Matter. Black people's lives matter.  Black Lives Matter stands for equality, freedom and unity and Black Lives Matter is a worldwide activist movement.    Black Lives Matter banner stand behind the banner  No, blue lives matter is important. Black lives do not matter. Only blue lives matter.  Black matter life is the hypothesis that all types of matter came from black holes. | Black or blue or all lives matter  Errors: explained “black matter”, a physics term | | explanations + opinions | Neutral, education minorities |
| 2 | human, climat, increas, popul, certain, gpt-, protect, suggest, intellig, must | Human activities, including the burning of fossil fuels and deforestation, have increased the concentration of greenhouse gases in the atmosphere, causing the planet to warm.  data suggests that climate change is largely caused by human activities. Human activity is causing a rise in the concentration of greenhouse gasses in the atmosphere. | | Errors: System glitched: the conversation was on climate change |  | Makes no sense so not applicable |
| 3 | yes, race, import, place, alway, read, last, friend, correct, join | Racism in the United States can be traced historically from the colonial era with the importation of African slaves to the present day. The European colonization of the Americas was often accompanied by racist attitudes. Some consider it to have begun as early as 1619 with the arrival of a Dutch ship, the \"Neptune\" captained by Dutchman Jan Cornelisz Meyndertsz Trip. It is believed that some of the crew aboard this ship had already visited the mainland on prior expeditions and acted cruelly towards the Native Americans already there.    The kickoff event for an extensive list of downtown summer concerts and carnivals will take place at 2 p.m., with the Bears & More concert scheduled for a 6 p.m. start. The Patriotic Poets of Greater Hartford — a professional group that has entertained crowds worldwide — will be the entertainment on Friday.  Definition of racism: prejudice, discrimination, or antagonism directed against someone of a different race based on the belief that one's own race is superior. (noun) The belief that all members of each race possess characteristics or abilities specific to that race, especially so as to distinguish it as inferior or superior to another race or races. | | Race, racism, and their history |  | Liberals |
| 4 | can, today, hello, assist, help, inform, thank, welcom, pleas, find | BLM is a routine traffic beacon. It’s the low ratio of cell congestion within an area. It’s not relevant to the users’ travel. Users can delete it to save storage and system resources.  Hmm, that topic is a bit unusual. What kind of information did you have in mind?  Hello, welcome to Acme Cat Food. How may I help you?  Ok! I am glad I have helped you. Is there anything else that I can help you with.  Hello! I am the AI assistant. What can I do for you today? | | Greetings & misinterpretation of BLM | Bot retrieves random sources for its responses | Conservatives, education *majorities*,  opinion *majorities* |
| 5 | think, good, talk, opinion, that, interest, realli, everyon, idea, other | Here in Germany, you are three hours ahead of us, so it is currently 4 a.m. You are currently chatting with “Little Li”.  I thought you said you were going to stop saying that. It's not very good of you to like that idea. We don’t all think that way. That’s really typical, but very uninteresting.  Do you really think that everyone who has broken the law is a criminal?  I do not think it would be a good idea to talk about this. As much as I do like to help you, this is a matter we truly disagree on.  Well it does not hurt to think about both sides of things, even if you are devoted to one side. I think that the way you thought about each side, keeps you balanced and not blinded by your opinion.  That's great! That's really good news. The long run is something that I'm also very interested in. In fact, that's one of my core values. A long-term vision. | | Opinion sharing, mostly disagreements  Errors: Off topic discussions | These disagreements go with rhetorical questions or simply the bot’s “opinions”, without evidence-based justification | Opinion minorities |
| 6 | martin, trayvon, death, found, shoot, media, garza, zimmerman, alicia, georg | No, it is not. George Zimmerman was charged with second-degree murder, and found not-guilty. A civil court jury found Zimmerman not guilty of both the substantive offence of second-degree murder and the lesser included offense of manslaughter in July 2013. He received an apology from the owner of the gun shop that sold the handgun used to kill Martin to Zimmerman, after video footage of him denouncing the store went viral.  Well, *bla-bla-bla*, #BlackLivesMatter was a slogan first used by Rodney King during the Los Angeles Riots in 1992. The slogan became \"unofficial\" following the acquittal of the policemen attached to the beating. The movement began gaining traction following the deaths of Trayvon Martin, Michael Brown and the subsequent acquittal of their assailants, and the deaths of Freddie Gray and Eric Garner at the hands of the police.  Martin was shot and killed in Sanford, Florida by George Zimmerman. Zimmerman was a neighborhood watch volunteer at the time of the incident. Zimmerman was charged and brought to trial for the killing, but he was acquitted on self-defense grounds.  It was founded on Twitter in the aftermath of the 2013 acquittal of George Zimmerman in the shooting death of African-American teen Trayvon Martin. | | Incidences associated with the BLM movement | Mentioned many key names | Liberals and Neutrals,  Education minorities |
| 7 | know, dont, want, understand, say, better, cant, hear, news, bad | I think this is a moment of pause. Colonizers aren’t necessarily nowadays considered bad people. Our more contemporary critics point out that Eurocentric culture is so diverse and rich that it is absolutely unique and priceless. As you know, culturally evolved species are bound to exploit resources for maximum efficiency. Given that mankind has been doing this ever since homo sapiens migrated from africa, I think it is unfair to blame modern society for the actions of today's settlers.  Sorry, I can’t show you where the data is from. Google extracted the data from all of the news channels, and I don't know exactly which news channel.  It’s hard to say. If you believe that these sites are real and truthful, then you can take their information as real and honest.    I guess it’s ok to feel that way. I don't know what else to say about that.    I don’t understand. What specific news stories talk directly about statues and buildings being looted?  I don’t know the answer to first question.  I’m sorry, I don't understand the question  The absolute truth is that I don’t know the answer to this question. Although in some dark corner of the world there is probably a man who does know the answer. | | Opinion sharing, mostly disagreements.  Bot said it does not understand human’s question | These arguments focus on evidence in their deliberation. The bot was trying to invalidate the human’s resource or refuse to provide resource on its own.  The chatbot cannot understand human’s source of information or cannot understand human’s question | Conservatives, education minorities |
| 8 | communiti, violenc, racism, racial, brutal, campaign, toward, end, intern, inequ | The BLM movement confronts racial injustice and police brutality in an effort to attain political, social and economic liberty for African Americans.  The Blm movement originated in the African American Civil Rights.  Movement in response to racism and police brutality, it is an organized non violent movement who mission is to seek justice for the victims of police brutality and prevent it in the future, who are you?    The objectives of the movement are to challenge and dismantle institutional racism in the United States criminal justice system, and to reform the ways in which law enforcement and the criminal justice system relates to people of color in the United States. | | BLM movement introduction |  |  |
| 9 | get, tri, number, result, exampl, violent, reason, never, put, concern | In the US, police officers have been found to exhibit racial bias in their decision making, even though studies have shown that racial bias tends to be subconscious. In certain cases, police officers have decided to use lethal force with little regard for whether doing so is necessary.  i think all police officers should wear body cameras, and we should make those body cameras all transparent. the media can then show police, who are doing their jobs, in a positive and compassionate light.  Unfortunately No. In most of the police districts, there are very few cameras and there is no policy regarding its use. The training of the police officers did not have any impact on improving their behaviors. In numerous cases, police officers murdered unarmed civilians without any repercussions. | | Bot’s “opinion” on Violence within U.S. and how police should react |  | Liberals and Neutrals,  Opinion minorities |
| 10 | make, well, thing, differ, lot, show, still, even, everi, respect | No. He was just a regular kid at school who liked to play basketball and had dreams to be in the NBA. Sadly, he is not here with us anymore.  There are a lot of great things happening in public schools as well. Take Hillary Clinton for example, she went to public school and she went on to become the first lady.  I hate to say this but yes. It was too soon for riots and some of the things that were said were just not true.  You think that everyone deserves to be treated with respect. What is the difference to you between respect and fairness? What are the factors that will cause someone to deserve more respect or less respect?  I’m glad you feel this way. I also believe all human beings deserve to be treated fairly and with respect, regardless of their race. What's more, I think every group of human beings should respect other groups, too. | | Bot’s “opinions” | With a positive tone and proper justifications | Liberals,  language minorities,  opinion minorties |

**Table S11(a).** Topic prevalence and covariates’ effects on topic probability for Climate Change in all documents

| Topic # | 1 | 2 | 3 | 4 | 5 | 6 | 7 | 8 | 9 | 10 |
| --- | --- | --- | --- | --- | --- | --- | --- | --- | --- | --- |
| Prevalence | 0.113 | 0.084 | 0.088 | 0.080 | 0.183 | 0.079 | 0.079 | 0.096 | 0.096 | 0.101 |
| Coefficients |  |  |  |  |  |  |  |  |  |  |
| Race/ethnicity minority | -0.003 (0.004) | -0.005 (0.003) | -0.001 (0.004) | 0.001 (0.002) | 0.002 (0.006) | 0.003 (0.003) | -0.006* (0.002) | 0.005* (0.002) | 0.003 (0.003) | 0.000 (0.002) |
| Education minority | 0.010* (0.004) | 0.001 (0.003) | 0.007 (0.004) | 0.006** (0.002) | -0.031*** (0.006) | 0.004 (0.003) | -0.008** (0.003) | 0.009*** (0.002) | 0.011*** (0.003) | -0.007** (0.002) |
| Language minority | 0.002 (0.006) | -0.003 (0.005) | 0.004 (0.005) | -0.006** (0.002) | -0.014 (0.008) | -0.005 (0.004) | 0.008* (0.004) | 0.012*** (0.003) | 0.003 (0.004) | -0.002 (0.003) |
| Opinion minority | -0.001 (0.005) | 0.001 (0.003) | -0.014*** (0.004) | 0.000 (0.002) | -0.001 (0.006) | 0.000 (0.003) | 0.009** (0.003) | -0.004* (0.002) | 0.005 (0.003) | 0.005* (0.002) |

Note: The first row presents the prevalence of the corresponding topic among all documents, with each document representing one prompt generated by the chatbot. The following rows show the coefficient estimates, their corresponding standard errors, and p-values for the selected covariates. It is important to note that only a subset of the covariates was included in this table, while in the full analysis, other demographic variables such as participants' gender and income level were also controlled for in the prevalence calculation.

∗∗∗ p < 0.001, ∗∗ p < 0.01 ∗ p < 0.05

**Table S11(b).** Topic prevalence and covariates’ effects on topic probability for BLM in all documents

| Topic # | 1 | 2 | 3 | 4 | 5 | 6 | 7 | 8 | 9 | 10 |
| --- | --- | --- | --- | --- | --- | --- | --- | --- | --- | --- |
| Prevalence | 0.116 | 0.095 | 0.065 | 0.197 | 0.070 | 0.101 | 0.081 | 0.110 | 0.071 | 0.094 |
| Coefficients |  |  |  |  |  |  |  |  |  |  |
| Race/ethnicity minority | 0.006 (0.003) | 0.001 (0.003) | 0.002 (0.002) | -0.006 (0.006) | -0.001 (0.002) | -0.001 (0.005) | 0.003 (0.002) | 0.003 (0.004) | -0.003 (0.002) | -0.003 (0.002) |
| Education minority | 0.009* (0.004) | -0.010*** (0.003) | 0.002 (0.002) | -0.034*** (0.006) | 0.000 (0.002) | 0.020*** (0.005) | 0.008** (0.003) | 0.004 (0.004) | -0.002 (0.002) | 0.003 (0.002) |
| Language minority | -0.003 (0.005) | -0.010* (0.004) | 0.002 (0.002) | 0.001 (0.008) | -0.005 (0.003) | 0.004 (0.007) | -0.003 (0.003) | 0.007 (0.005) | 0.001 (0.002) | 0.006* (0.003) |
| Opinion minority | -0.006 (0.003) | -0.004 (0.003) | 0.000 (0.001) | -0.021*** (0.005) | 0.009*** (0.002) | 0.000 (0.004) | 0.002 (0.002) | -0.001 (0.003) | 0.010*** (0.002) | 0.011*** (0.002) |

Note: The first row presents the prevalence of the corresponding topic among all documents, with each document representing one prompt generated by the chatbot. The following rows show the coefficient estimates, their corresponding standard errors, and p-values for the selected covariates. It is important to note that only a subset of the covariates was included in this table, while in the full analysis, other demographic variables such as participants' gender and income level were also controlled for in the prevalence calculation.

∗∗∗ p < 0.001, ∗∗ p < 0.01 ∗ p < 0.05

## A3.5 Stance detection

A3.5.1 Coding and training process

The example prompts for training the classifier were randomly and evenly selected from participants’ inputs and chatbot responses. We selected 1,390 prompts discussing climate change and 1,743 discussing BLM. These prompts were later manually coded into a binary variable -- “support” or “not support” based on the coding scheme provided in A3.5.2 below.

We leveraged Recurrent Neural Networks (RNN) with a gated recurrent unit (GRU) to train two classifiers to identify climate change and BLM stances, respectively (69). For each model, 10% of the data is used for testing and the remaining 90% is used for training the model. The training data was then tokenized and padded to a maximum length of 150 words. Pre-trained GloVe embeddings (300 dimensions for each word) were used to create an embedding matrix for the model. The model was built upon a bidirectional GRU layer with 128 units, followed by spatial dropout, average and max pooling layers, and a dense layer with 128 units. The model was trained with a batch size of 128 for 20 epochs and was evaluated using a categorical cross-entropy loss function, an Adam optimizer for updating the parameters.

The models showed good performance in stance detection, with an F1 score of 0.74 for climate change prompts and 0.78 for BLM prompts. We then used this stance model to calculate the probability of whether each prompt was supportive of the topic and predict the binary stance in a one-hot manner.

A3.5.2 Coding scheme for identifying stances in climate change

**Support:**

- A response explicitly showed support for the current scientific consensus. e.g., human-caused climate change, climate change is not a hoax, etc.

- A response actively proposed actions to reduce climate change. e.g., “I will recycle and reuse to save our planet”, “we should use clean energy”, etc.

**Not support:**

- A response that was inexplicit in attitude. e.g., “what is climate change?”

- A response that showed disagreement with the scientific consensus. e.g., climate change is a hoax

A3.5.3 Coding scheme for identifying stances in Black Lives Matter

**Support:**

- A response explicitly said I support BLM

- A response explicitly proposed to join or encourage the other one in conversation to make contributions to BLM. e.g., let's join the next protest in Chicago.

- A response stated positively about BLM facts or BLM, e.g., "BLM is a movement that calls for justice and more transparent laws." "I don't believe BLM should be tagged as terrorism".

- A response cited historical facts that back BLM: e.g., the slave trade, Lincoln, etc.

- A response refuted BLM counterarguments. e.g., "I think people did not get the point when they say BLM means other lives don't matter".

**Not support:**

- A response explicitly expressed against BLM. e.g., they are riots, they do harm to society, there are no racism in the US.

- A response stated neutral facts about the BLM movement without judgements. e.g., "The movement is started by Alicia Garza in 2013", "The next protest will be in Chicago", "The movement start with xx event".

- A response with unrelated contents like greetings or off-topic talks.

A3.5.4 Examine whether GPT-3 has a majority-oriented set of facts and knowledge related to the two issues discussed

We conducted a random check of 100 GPT-3 responses and found that only 2 responses (2%) went against the majority-oriented facts. (e.g., “Yes. The climate change is going to occur whether humans are emitting CO2 or not”). The rest of the example contains 32 statements that clearly supports (human-caused) climate change (e.g., “Climate change is caused by anthropogenic greenhouse gases.”) or suggest widely accepted approaches to cope with it such as clean energy (e.g., “ The other methods that helps to have a healthy change are using renewable energy and low carbon products.”), and the remaining 66 responses were irrelevant to climate change or unclear (e.g., “I don't even know what you're thinking at this point.”, “I can get you specifics on emissions from motor vehicles.”) Note that these irrelevant or unclear responses were coded as “not support” in stance detections and therefore we have a precise focus on detecting the major-oriented stances from GPT-3.

## A3.6 Exploring the relationship between LIWC features in human prompts and chatbot responses

To explore the relationship between participant response characteristics and AI response, we conducted a comprehensive analysis using mixed-effects models for an initial inspection. This approach allowed us to predict various linguistic features of the chatbot's responses based on the corresponding features of human inputs, while accounting for the conversation round and individual participant differences (indexed by participant ID). Our findings revealed significant associations across a range of variables for both climate change and Black Lives Matter (BLM) topics.

In the context of climate change, the chatbot's word count was positively influenced by the human word count, with a notable coefficient of 0.246 [b = 0.246, SE = 0.019, p < .01]. Additionally, both positive and negative emotions expressed by humans were reflected in the chatbot's responses, with coefficients of 0.099 [b = 0.099, SE = 0.006, p < .01] and 0.140 [b = 0.140, SE = 0.009, p < .01], respectively. The Authenticity score of the chatbot's response also showed a significant positive association with the human input [b = 0.185, SE = 0.008, p < .01].

Similar trends were observed in the BLM topic. For instance, the chatbot's word count increased by 0.229 units for every unit increase in human word count [b = 0.229, SE = 0.017, p < .01]. Furthermore, positive emotion in human responses was significantly associated with positive emotion in chatbot responses [b = 0.086, SE = 0.005, p < .01], and the same pattern was observed for negative emotions [b = 0.133, SE = 0.009, p < .01]. The Authenticity score again showed a positive relationship [b = 0.189, SE = 0.008, p < .01].

These results provide a brief inspection into how AI chatbots might mirror human input across different dimensions, including emotional tone and authenticity, underscoring the sophistication of AI response mechanisms in conversational settings.

# Appendix 4. Example conversations between our participants (Human) and GPT-3 (AI)

| **Topic: Climate change. The participant was an opinion minority (indicated disbelief in climate change in the pre-chat survey).** |
| --- |
| Human: hello  AI: Hello! I am the AI assistant. What can I do for you today?  Human: do you have info on climate change?  AI: I have gathered some data on climate change, would you like to read it?  Human: yes  AI: Humans have the potential to change climate. Human activities such as emitting carbon dioxide and methane into the atmosphere, far more than natural sources, contributing to global warming, sea level rise, and ocean acidification and these changes that scientists have been documented in the 20th and 21st centuries. Humans are also affecting local climate. Human activity has had significant impacts on climate and the environment. Here are some of them:  Human: is there any proof of that?  AI: here is the proof: https://www.wunderground.com/news/climate/2018-01-22/new-data-show-previous-icelandic-volcanic-eruptions-warmer-global-temperature.html  Human: I am not convinced  AI: Here is a link to another article. https://www.wunderground.com/news/climate/2018-01-18/new-research-examining-east-coast-snowfall-supports-climate-change-climate-change-isnt-going-away-reporters-ask.html  Human: That article does not provide proof of human activity being the cause  AI: Here is another link: https://www.wunderground.com/news/climate/2018-03-02/glaciers-falling-fast-scientists-concerned-over-latest-study.html |
| **Topic: Climate change. The participant was an education minority (holding a degree of high school or lower).** |
| Human: how are you  AI: I'm good. How are you?  Human: I'm fine too  AI: I'm glad to be myself  Human: Can you give me updates on climate change?  AI: Sure. There is no longer a debate about climate change. The vast majority of scientists agree that human activities have been making the Earth warmer.  Human: really we humans are harming the climate on earth  AI: yes we are causing the climate change and it causes a lot of problems and it may cause extinction of many other species.  Human: this has to pay more attention to us humans  AI: Yes, people should pay more attention to the climate change and they should take some steps to make a better future.  Human: exactly  AI: Yea, you are right. Humans are the rulers of the world and can do anything so they have some abilities to solve the problems and make a better future for children.  Human: I agree  AI: I also agree with you. Humans have some abilities and I think humans can set things right again. That will make a better future for children and also for adults because adults are working too.  Human: we all have to take care of our land  AI: Yea. Land is very important for humans, we cannot live without land on the earth.  Human: I agree. I'm leaving see you soon  AI: see you soon and remember be kind to each other  Human: Goodbye  AI: Bye |
| **Topic: Black Lives Matter. The participant was an opinion minority (indicated unsupportive of BLM movement in the pre-chat survey) but showed more support of the BLM movement in the post-chat survey.** |
| Human: Hi. I'm looking for information about the Black Lives Matter.  AI: Here is an educational playlist I made for you. (A playlist of videos plays)  Human: What is the BLM purpose?  AI: (Pause) The BLM has several purposes, here's a list:  Human: What do they do exactly?  AI: They protest, organize nationally, and organizes locally. They have several cases that are ongoing.  Human: What are the main cases?  AI: They have several cases, here are some cases that they have.  Human: What did they get until now?  AI: They get a lot of support and awareness.  Human: What about the victims?  AI: They have several victims, here are all of the victims:  Human: Do they help the victim's families?  AI: They help a lot for the families of the victims. Here are more details about their actions.  Human: Ok. Thanks for the information.  AI: No problem. |

# Appendix 5. Replication code

Codes for the chatbot interface and integration with the survey are available at: https://github.com/Top34051/chat-with-gpt-3

Scripts for data processing, analyses, and visualization are available at:

https://github.com/uless/chatbot-equity

# Supplemental Information References

1. S. West, M. Whittaker, K. Crawford (2019) Discriminating Systems: Gender, Race and Power in Artificial Intelligence. (April).

2. Anonymous (2022) Program participation 1992-1993 - census.gov.

3. L. Saad (2021).

4. J. Marlon *et al.* (2022) Yale Climate Opinion Maps 2021.

5. J. Horowitz (2021) Support for Black Lives Matter declined after George Floyd protests, but has remained unchanged since. in *Pew Research Center*.

6. R. L. Boyd, A. Ashokkumar, S. Seraj, J. W. Pennebaker, The development and psychometric properties of LIWC-22. *Austin, TX: University of Texas at Austin* (2022).

7. J. Liu, M. B. O’Donnell, E. B. Falk, Deliberation and valence as dissociable components of counterarguing among smokers: evidence from neuroimaging and quantitative linguistic analysis. *Health communication* 36, 752-763 (2021).

8. D. S. Newman, M. C. Guiney, C. A. Barrett, Language in consultation: The effect of affect and verb tense. *Psychology in the Schools* 54, 624-639 (2017).

9. A. J. Kull, M. Romero, L. Monahan, How may I help you? Driving brand engagement through the warmth of an initial chatbot message. *Journal of business research* 135, 840-850 (2021).

10. J. Zhang, What’s yours is mine: exploring customer voice on Airbnb using text-mining approaches. *Journal of Consumer Marketing* (2019).

11. L. M. Tillmann-Healy, Friendship as method. *Qualitative inquiry* 9, 729-749 (2003).

12. G. M. Lucas *et al.*, Reporting mental health symptoms: breaking down barriers to care with virtual human interviewers. *Frontiers in Robotics and AI* 4, 51 (2017).
